# Rhythmic modulation of prediction errors: A top-down gating role for the beta-range in speech processing

**DOI:** 10.1371/journal.pcbi.1011595

**Published:** 2023-11-07

**Authors:** Sevada Hovsepyan, Itsaso Olasagasti, Anne-Lise Giraud

**Affiliations:** 1 Department of Basic Neurosciences, University of Geneva, Biotech Campus, Genève, Switzerland; 2 Institut Pasteur, Université Paris Cité, Inserm, Institut de l’Audition, France; Brain and Spine Institute (ICM), FRANCE

## Abstract

Natural speech perception requires processing the ongoing acoustic input while keeping in mind the preceding one and predicting the next. This complex computational problem could be handled by a dynamic multi-timescale hierarchical inferential process that coordinates the information flow up and down the language network hierarchy. Using a predictive coding computational model (Precoss-β) that identifies online individual syllables from continuous speech, we address the advantage of a rhythmic modulation of up and down information flows, and whether beta oscillations could be optimal for this. In the model, and consistent with experimental data, theta and low-gamma neural frequency scales ensure syllable-tracking and phoneme-level speech encoding, respectively, while the beta rhythm is associated with inferential processes. We show that a rhythmic alternation of bottom-up and top-down processing regimes improves syllable recognition, and that optimal efficacy is reached when the alternation of bottom-up and top-down regimes, via oscillating prediction error precisions, is in the beta range (around 20–30 Hz). These results not only demonstrate the advantage of a rhythmic alternation of up- and down-going information, but also that the low-beta range is optimal given sensory analysis at theta and low-gamma scales. While specific to speech processing, the notion of alternating bottom-up and top-down processes with frequency multiplexing might generalize to other cognitive architectures.

## Author summary

During speech perception, our brain achieves *continuous* acoustic analysis of the ongoing speech signal, its transformation into linguistic representations, and the prediction of the most likely next words or syllables. In this computational study, we address the biological mechanisms underpinning the coordination of these operations during natural speech processing. Using a model that recognizes on-line syllables in natural sentences, we show that neural activity at specific rhythms is dedicated to specific operations, and that while the theta and low-gamma rhythms are engaged in speech features signaling and encoding, the more endogenous low-beta rhythm drives the rhythmic and coordinated modulation of prediction errors across levels of hierarchy.

## Introduction

A key challenge in speech processing is the ability to analyze what has just been said while processing what is being said and predicting what will follow, the so-called “now or never bottleneck” [1]. This threefold challenge does not only require an appropriate neural architecture but also an efficient temporal orchestration of the neural event sequence involved, allowing through an inferential process for joint information intake, processing and prediction. During this inferential process takes place in a left-hemispheric network [2–4] where information flows up and down the hierarchy via feedforward and feedback connections and spreads at each stage via lateral connections [5–7]. Speech recognition results from the precise interplay between these feedforward, feedback and lateral streams during the multi-level inference [8–10]. Whether the inferential process involves continuous or discrete/alternating operations, and at which rate(s) they possibly occur is an essential piece of the puzzle.

Neural oscillations, as a proxy of rhythmic collective neuronal activity [11–13], are directly involved in various aspects of speech processing [14,15], including speech chunking at different granularity levels depending on their frequency (phrases, words, syllables, phonemic features) and information encoding depending on their cross-frequency interactions [16–20]. Theta (4-7Hz) and low-gamma (25-35Hz) oscillations are related to bottom-up processes, notably the hierarchical encoding of phonemic information within syllables [17,21,22]. Delta (1-4Hz) and low-beta (14-21Hz) oscillations, which are also frequently observed in relation with speech processing, have a more endogenous origin. While delta is argued to play a role in syntactic parsing [23,24], beta (15-30Hz) oscillations are associated with comprehension and top-down effects, without having been hitherto related to specific linguistic units or language operations [10,25–28].

The notions of neural oscillations and hierarchical inference are likely intimately related to cognitive processes, notably in speech reception [6,26,29,30]. Experimental studies and theoretical proposals suggest that information is generally transferred up and down the hierarchy using different frequency channels [29,31–33]. Gamma oscillations (30-100Hz) are related to bottom-up information and prediction errors, i.e. the discrepancy between cognitive expectations and sensory signals [33–35], whereas beta oscillations (15-30Hz) are rather associated with top-down predictions and modulatory signals [32,33,36,37]. The exact computational function of the latter, however, and their possible interplay with upgoing signals remains unclear [31,38–42].

Several hypotheses have nevertheless been formulated [42–44]. Beta could work as an information channel conveying predictions down the processing hierarchy [45,46], or, according to the predictive routing hypothesis, it could also prepare specific pathways by inhibiting neural populations that encode expected sensory signals, lowering the processing cost of novel information [36,47]. Not incompatibly, it might also reflect the delay for integrating bottom-up sensory signals and updating predictions [29]. In the same vein, recent work suggests that beta oscillations could directly be related to the weighting of sensory prediction errors [48].

Following-up on this, we used computational modeling to address the possible function of beta oscillations in the rhythmic weighting of prediction error in the context of speech processing. We built on a previous model that uses theta (~5Hz) / gamma (~40Hz) oscillation coupling in a predictive coding framework to achieve natural speech parsing and *on-line* syllable identification in continuous natural speech [49]. In the new model, *Precoss-β*, we explore how alternating top-down and bottom-up information streams via the rhythmic weighting of prediction errors affects the inference process.

Modulating prediction error precisions (PEP) within a frequency range spanning from 2 Hz to 60 Hz, for both syllable identity and timing, we found that *Precoss-β* outperforms its previous version with non-modulated prediction errors, and is most efficient when precisions are modulated at the beta range (20-30Hz). These results suggest that the low-beta rhythm could support online speech recognition by controlling the alternation of a bottom-up versus top-down dominant mode during the inference process. The observed benefit reflects that the model can flexibly pick up unexpected input while remaining both sensitive to bottom-up information and reliable in terms of predictions, hence achieving the triple challenge of speech processing.

## Results

### *Precoss-β* architecture and oscillating precisions

**Fig 1:**
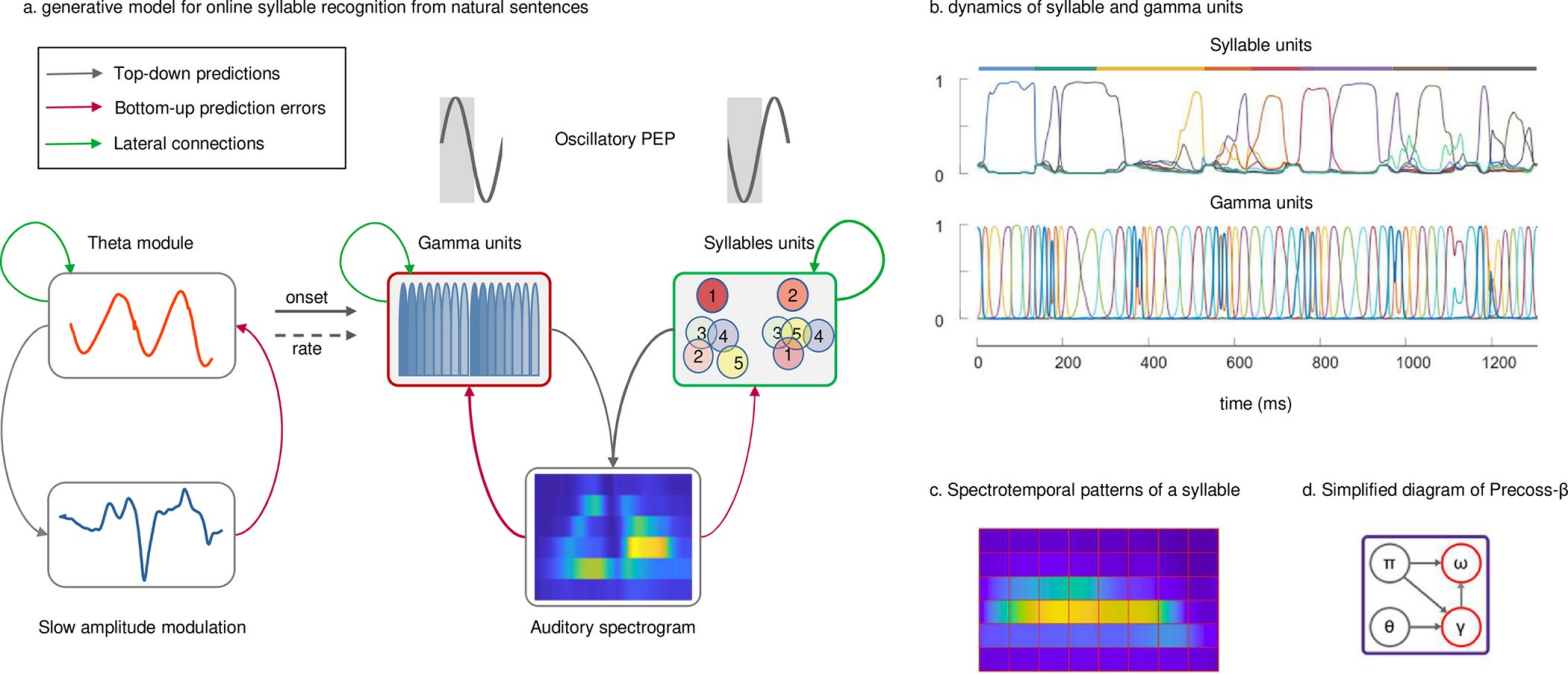
A generative model for on-line syllable recognition with rhythmic state-dependent precisions. The diagram in subpanel a) shows the simplified schematics and hierarchical message passing of Precoss-β. The lower panel shows the input to the model. As in the original model (Precoss), the input consists of the speech slow amplitude modulation (on the left) and the auditory spectrogram (on the right). At the top level, the theta module tracks the slow amplitude modulation in the input and feeds it to a theta oscillator. The instantaneous frequency of the theta oscillator and the Gaussian pulses associated with the predefined phases signal speech rate and syllable onset information to the gamma units. Together, the gamma and syllable units produce the auditory spectrogram in the input, based on the spectrotemporal patterns stored in the model’s memory (subpanel c). The gamma units make temporal predictions about the spectrotemporal patterns of syllables (as many as there are syllables in the input sentence), while the syllable units accumulate evidence about each syllable in the sentence. Depending on the phase of the oscillating PEP, the model changes the precision of the syllable and gamma units, modulating the influence of the corresponding prediction errors on the dynamics of the hidden states. Depending on the phase of the precision units (highlighted by the grey rectangle), either syllable or gamma units get higher precision. The arrows represent a message passing between levels of the model hierarchy (top-down predictions in grey and bottom-up prediction errors in magenta) and lateral, within-level connections (green arrows). Subpanel b) shows an example of the dynamics of syllable and gamma units from the model simulations. The top panel shows the accumulated evidence for different syllables (color coded) in the input sentence (colored bars on top of syllable units represent syllables in the input sentence), while the bottom panel shows the sequential activation of gamma units. Subpanel d) shows the simplified diagram of the model, where θ, γ and ω represent theta module, gamma and syllable units respectively. The π represents the oscillating precision (arrows indicate the units whose precision is controlled).

*Precoss-β* was built by including oscillating state-dependent precisions within a previously described generative model [49] that parses and identifies syllables from continuous speech. The model input consists of a speech reduced auditory spectrogram [50] and of its slow amplitude modulations [17], both extracted from English sentences of the TIMIT database [51] (see Hovsepyan et al. 2020 [49] for details about speech input generation). In *Precoss*, the activation of the appropriate syllable unit generates the corresponding auditory spectrogram with a flexible duration determined by eight gamma units (Fig 1A). Syllable and gamma units represent syllable identity and timing within the syllable, respectively. Together with the other model elements, they serve to deploy predictions (grey arrows) about the input acoustic spectrogram. The ongoing mismatch (red arrows) between predicted and actual auditory spectrograms and slow amplitude modulations drives the inference process across the model hierarchy and leads to updating syllable and gamma units (Fig 1B), as well as all other variables in the model, such that predictions best match the input.

As our goal is to assess how rhythmic fluctuations of internal expectation vs. bottom-up prediction errors drive the model updates with respect to syllable identity (syllable units) and timing (gamma units), and affect performance, we introduced specific units that control the precision of syllable and/or gamma units (variants *Precoss-β*-*identity*, *Precoss-β*-*timing* and *Precoss-β*-*full*). These precision units effectively modulate the relative strength of internal predictions based on previous time points and bottom-up prediction errors in the updates of syllable and/or gamma units. This is qualitatively different from the previous model with fixed precisions and affords a new degree of flexibility.

The model performance is assessed based on the output of syllable units (Fig 1B), which summarizes the model estimate about the syllable boundaries and identity in the speech input. Performance metrics are based on comparing the estimated syllable sequence with the one actually present in the input (S1 Fig).

### Model variants and performance

To assess the effect of modulating top-down and bottom-up information streams, we compared the performance of *Precoss* (stationary precisions) and *Precoss-β* (oscillating precisions) in their ability to parse and recognize syllables from natural spoken sentences. Whatever the model version, the input is a full natural sentence without explicit syllable boundaries. The model parses it into discrete units and identifies the sequence of activated syllables.

Since predictions about the auditory spectrogram (the input) are generated in concert by syllable units that recognize the overall spectrotemporal pattern, and gamma units that specify the position of the acoustic segment within the overall pattern, the discrepancy between the predicted and actual input can in principle be solved by updating both the estimate of *where* we are in the pattern (gamma units) and the pattern identity (*what*–syllable units).

**Fig 2:**
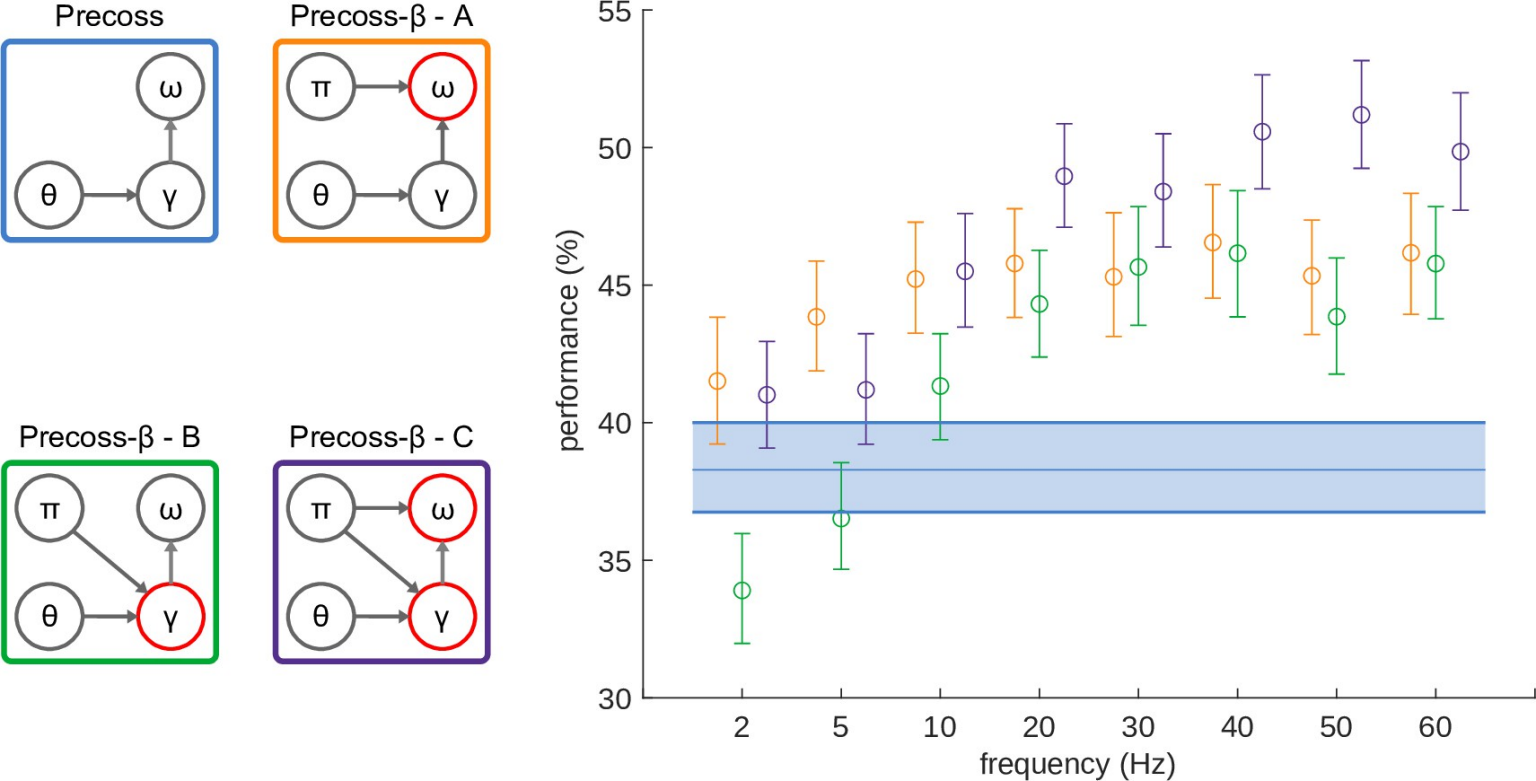
Model performance based on the overlap measure. We tested the online syllable recognition accuracy of the model based on simulation results on 220 sentences (giving a total of about 3000 syllables). Accuracy was evaluated based on the overlap of the recognized syllable sequence and durations with those of the input sentence. Data for each model variant is represented by the color of the outlines on the left panel. The figure shows the mean performance and 95% confidence interval for each frequency value of precision units. Diagrams on the left indicate the main functional groups of the model: θ corresponds to the theta-module, γ and ⍵ to syllable and gamma units respectively. Arrows indicate connections between functional groups (θ → γ represents rate and onset information from theta module to gamma units, whereas γ → ⍵ indicates the reset of accumulated evidence by the last gamma unit). π represents precision units, and the arrows originating from it indicate which functional groups they control.

We therefore run simulations varying the frequency at which precision units modulate syllable and gamma units. We compared model variants (Fig 2, left panel) where oscillating precisions drive: causal syllable units alone (*Precoss-β-identity*), causal gamma units alone (*Precoss-β-timing*), or both in anti-phase (*Precoss-β-full*). In the latter case, anti-phase refers to the fact that when syllable units are in a high precision state, gamma units are in a low precision one, and vice versa (Fig 1A). We also considered the case where both causal gamma and syllable units are in phase (*Precoss-β-full-samephase*, Fig 4). The original model with stationary precisions provides baseline performance. The simulations were run on the same set of 220 natural sentences.

We posit that modulating the relative strength of internal expectation and bottom-up information in a rhythmic fashion should improve performance as it alternatively sensitizes the model to internal knowledge vs. external evidence, which, given the altogether predictive and fluctuating nature of speech, should be an optimal processing strategy. We also expected the model performance to depend on the prediction error precision rate, peaking at a frequency that will depend on the two model intrinsic rhythms (~ 5 Hz for syllable units, ~ 40 Hz for gamma units).

In *Precoss-β*-identity (A) PEP are only modulated in the syllable units, which act as evidence accumulators for each syllable in the input sentence. Therefore, to benefit from the alternation between top-down and bottom-up information flows on the inference process, there should be at least one full PEP cycle per syllable. As the mean syllable duration in our dataset is around 200ms, we anticipate the preferred PEP modulation frequency to lie within the theta range ~ 5 Hz.

Similarly, in *Precoss-β*-timing (B) PEP are only modulated for the gamma units. Those units are responsible for deploying spectrotemporal predictions at the right time and in the correct order. They operate at gamma scale (40 Hz, at rest). With the same logic as for *Precoss-β*-identity, we expect a positive effect on alternation to require a PEP modulation frequency within the gamma range.

Finally, in *Precoss-β*-full (C) PEP are modulated in both syllable and gamma functional groups. As information about syllable identity in the input fluctuates at the theta range and information about timing fluctuates at the higher gamma range, we expect the optimal common PEP frequency to lie somewhere between 5 Hz and 40 Hz.

Fig 2 shows the performance of *Precoss-β* variants together with that of the original *Precoss* with stationary precisions. To quantify syllable recognition performance, we compared the model output and input with a metric that takes into account both the order and duration of the syllables and varies between (0–100%) (for details about this metric see S1 Fig). For almost all conditions, *Precoss-β* (oscillating precisions) significantly (S1–S3 Tables) outperformed *Precoss* (stationary precisions). That is, the rhythmic alternation of internal expectations and bottom-up influence on the inference process improves online syllable recognition from natural sentences.

The orange dots and ranges represent the mean performance and 95% confidence intervals for *Precoss-β-identity* obtained by bootstrapping with 10000 reps. For all tested PEP modulation frequencies, *Precoss-β-identity* performed better (Wilcoxon signed rank test, Z = 4.89, p = 9.7634–7, at 5 Hz) than *Precoss* with stationary precisions (blue line). The difference was statistically significant (p<0.05) for all frequency values (except 2 Hz) (S1 Table). However, no optimal frequency arose; performance reached a plateau at 5Hz and fluctuations beyond 5Hz were not statistically significant (S2 Fig, S4 Table).

Simulation results for *Precoss-β*-timing are presented in green. Interestingly *Precoss-β* with oscillating precisions performed lower than *Precoss* with stationary precisions for low modulation frequencies (Wilcoxon signed rank test, Z = -3.382, p = 0.0007 at 2 Hz) and higher for modulations >10 Hz does (Wilcoxon signed rank test, Z = 4.568, p = 4.915e-6 at 20 Hz) (S2 Table). Although performance is higher in the gamma range (Wilcoxon signed rank test, Z = 5.4, p = 6.283e-8 at around 40Hz), pairwise comparisons were not statistically significant for frequencies equal or greater than 20 Hz, indicating a knee point at this frequency (S3 Fig, S5 Table).

Finally, *Precoss-β*-full, which controls precisions of both syllable and gamma units, outperformed *Precoss* for all frequency values (S3 Table). Here again, we do not see a preferred frequency for the best model performance, instead, performance increases with frequency and reaches a plateau at around 20 Hz (Wilcoxon signed rank test, Z = 8.22, p = 1.937e-16). While for lower frequencies *Precoss-β-identity* (A) and *Precoss-β-full* (C) perform similarly, for frequencies higher than 20 Hz, *Precoss-β-full* (C) outperforms the other model variants (N-Way ANOVA, F = 15.92, p = 0, S8 Table). As for *Precoss-β-full* (C), pairwise comparisons of model performance for different frequencies higher or equal to 20 Hz, were not statistically significant (S4 Fig, S6 Table).

Performance based on the overlap metric (Fig 2) depicts the ability of the models to correctly identify syllable identity in a categorical way, as well as to infer the correct syllable duration (S1 Fig). However, it does not take into account the uncertainty associated with the identified syllable (e.g., the difference in activation between the winning syllable and the second-best candidate within the gamma sequence-defined window). We therefore considered a modified overlap metric that was weighed by the entropy of the syllable hidden states within each gamma sequence-defined window. Based on this entropy weighted overlap metric, Precoss-β always outperforms Precoss with fixed precision (S7 Fig, S13–S15 Tables). However, across Precoss-β variants, the performance differences become less tangible (S7 Fig, S20 Table), the best performing model being *Precoss-β-identity*.

Furthermore, we also compared models based on the longest common subsequence metric (LCS) between recognized and input syllable sequences (S8 Fig and related S9 and S10 Tables). In contrast to the overlap-based metrics, the LCS is sensitive to the order of recognized syllables and does not depend on how well the model can infer syllable durations. With this metric Precoss-β outperforms Precoss with fixed precision only when the PEP frequency of Precoss-β-full is at least in the beta range (20 Hz) (S27 Table).

Finally, to account for the different variable complexity of Precoss (17 variables) and Precoss-β (19 variables), we calculated the Bayesian Information Criterion (BIC). S29 Table shows the BIC values for each Precoss-β for all PEP frequencies tested. Interestingly, Precoss-β variants have a higher BIC value when the oscillating PEP frequency is at least 10–20 Hz, except for Precoss-β-identity which has a higher BIC value than Precoss only for 50–60 Hz. Overall, these results suggest that oscillating PEP improves online syllable recognition, and that the improvement depends on the frequency of the PEP: a plateau is reached around the cortical beta range.

### Integration of bottom-up information

We next consider how the frequency of the oscillating PEP affects the model’s ability to integrate sensory information, specifically syllable identity information propagated up in the hierarchy via bottom-up prediction errors. We quantified this ability by how often the accumulated evidence about a syllable changed in the same direction as the prediction errors signaling the presence of that syllable in the input.

**Fig 3:**
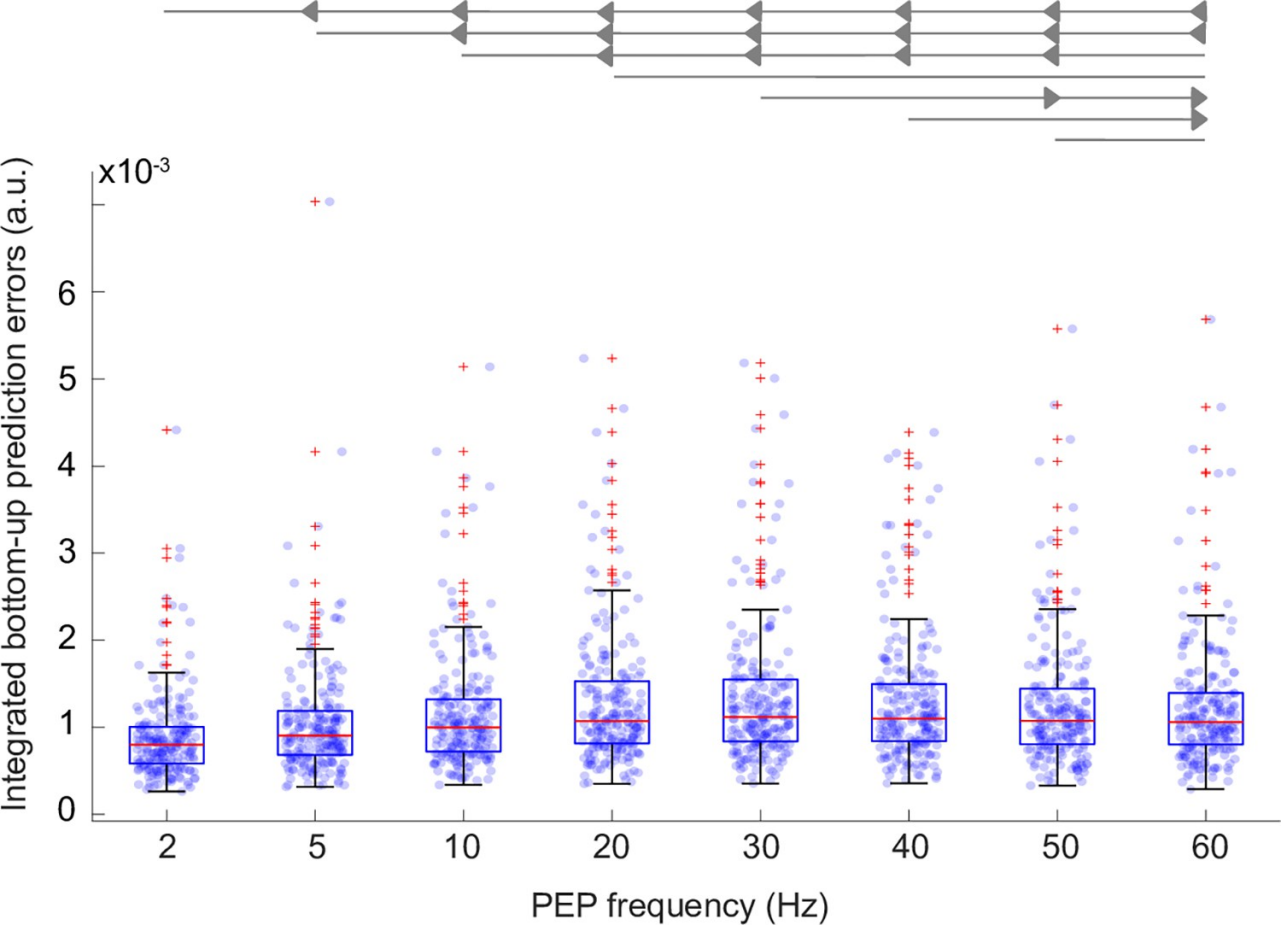
Sensory information integration efficacy—*Precoss-β-full*. We quantified how the modulation frequency of the PEP affects the model’s ability to integrate sensory information about syllable identity. The Friedman test indicated that the modulation frequency affected the amount of informative signal propagated up in the model hierarchy. Pairwise comparisons were made for each frequency pair (S12 Table). The Bonferroni procedure was used to control for multiple comparisons. The measure of the integration of sensory information peaked at 30 Hz, with statistically significant (p<0.05 corrected) differences from all other frequencies except 20 and 40 Hz. Each point on the scatter plot represents the measured value for each sentence at the corresponding PEP frequency. The scatter plots are overlayed with boxplots; the central red marker corresponds to the median, the lower and upper edges represent the 25th and 75th percentiles, and red crosses indicate outliers, while whiskers extend to the highest and lowest performance values that are not considered outliers. Arrows at the top indicate (following the convection described in [97]) a significant difference and direction of effect (left or right arrow) between the frequencies compared.

The results are presented in Fig 3. Frequency significantly affected the integration efficacy (Fig 3, Friedman test, χ^2^ = 269.85, p = 1.635e-54), which was statistically higher at 30 Hz than at all other frequencies except 20 and 40 Hz (Bonferroni corrected post-hoc pairwise comparisons, see S12 Table for details). These results suggest the beta range as an efficient modulation frequency for alternating the influence of top-down and bottom-up information.

### Effect of PEP modulation phase

Among the three model variants, the best performance (largest number of recognized syllables with the least uncertainty) is obtained for the one where PEP are modulated in both syllable and gamma units. By construction, *Precoss-β-full* controls the precisions of syllable and gamma units in opposite directions; whenever the precision of syllable units increases, the precision of gamma units decreases and vice versa. This choice was based on the idea that syllable units and gamma units can take turns in *absorbing* prediction errors, making it easier for the model to find the right estimates.

**Fig 4:**
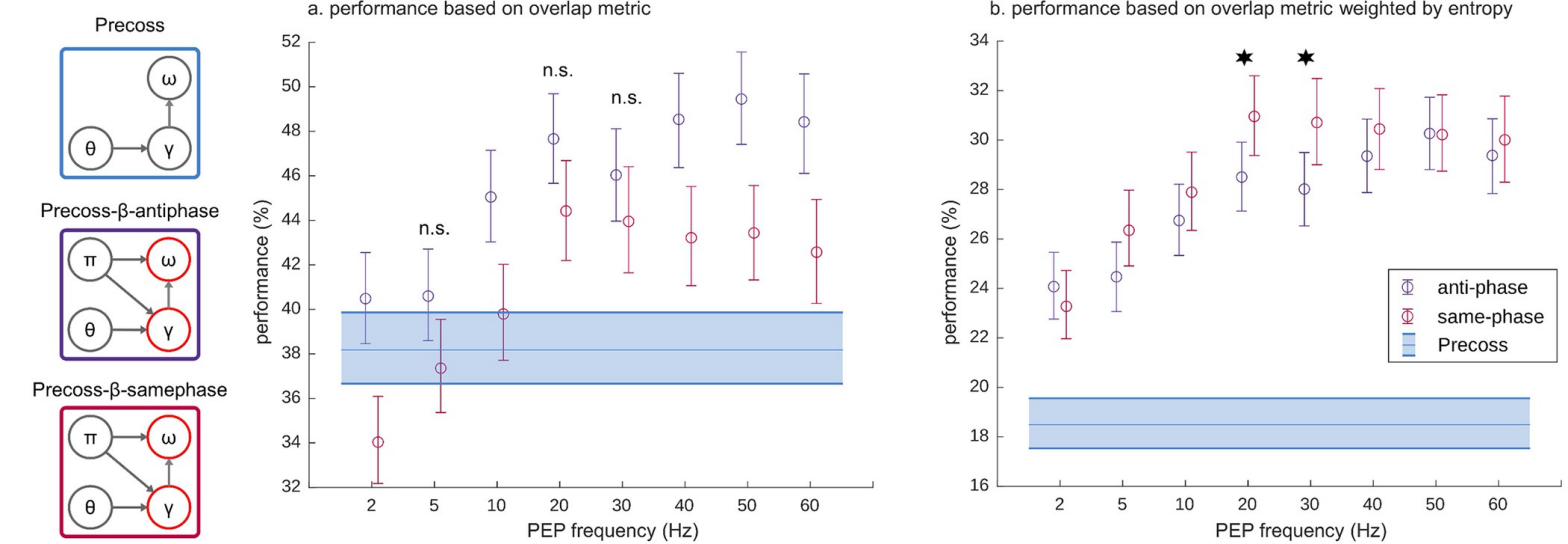
Effect of the oscillating PEP phase on model performance. *Precoss-β-antiphase* controls the precision of both syllable and gamma units so that the high precision state for syllable units coincides with the low precision state for gamma units (anti-phase condition, indigo). Here we tested whether the performance depends on the phase lag for the precisions of the syllable or gamma units. Therefore, we also tested *Precoss-β-samephase* when syllable and gamma units reach a high precision state simultaneously (same-phase condition, magenta). The left panel (a) evaluates the models based on the overlap metric, while the right panel (b) shows the performance of the models based on the entropy weighted overlap metric. In both plots, the means and corresponding 95% confidence intervals are shown.

To address how this *a priori* choice affected performance, we also run the model with precisions of gamma and syllable units oscillating in-phase (same-phase condition, Fig 4A red). On the one hand, the model with anti-phase condition outperformed the model with same-phase conditions in statistically significant manner at most PEP frequencies for our first overlap metric (Fig 4A, S9 Table). This finding shows that the model performs better when bottom-up prediction errors are preferentially minimized in alternation by syllable and gamma units, when syllable identity and timing features are analyzed via concurrent streams. Interestingly, when the PEP frequency was in the beta range (20–30 Hz), the difference in performance was not statistically significant. On the other hand, when considering the entropy-weighted overlap metric, the difference between same-phase and anti-phase vanishes (Fig 4B, S9 Table), except for the beta range, where same-phase outperforms anti-phase. Thus, except in the beta region where the preference for same-phase condition leads to slightly better performance, the model’s performance benefits from the rhythmic, sequential alternation of the PEP of both syllable and gamma units.

## Discussion

The goal of this study was to explore the possible role of cortical beta oscillations in speech processing from a theoretical perspective, where the brain deploys predictions through top-down and lateral connections and refines them based on bottom-up prediction errors [5,52,53]. Here, we conjectured that beta oscillations might set the alternation of bottom-up versus top-down control in the brain’s inference process. We tested this hypothesis by introducing precisions that oscillated in time within specific functional groups (syllable recognition and timing units) and comparing performance across frequencies with a baseline/control model with stationary precisions. We found oscillating PEP improved performance relative to stationary PEP; the alternation allowed the model to react to changes in the input without compromising the strength of top-down predictions, which lead to both more accurate and more precise inference. This contrasts with stationary PEP, which renders the model either too reactive to input when precisions are low, or too inflexible when precisions are high.

### The added value of rhythmic prediction error precisions (PEP)

**Fig 5:**
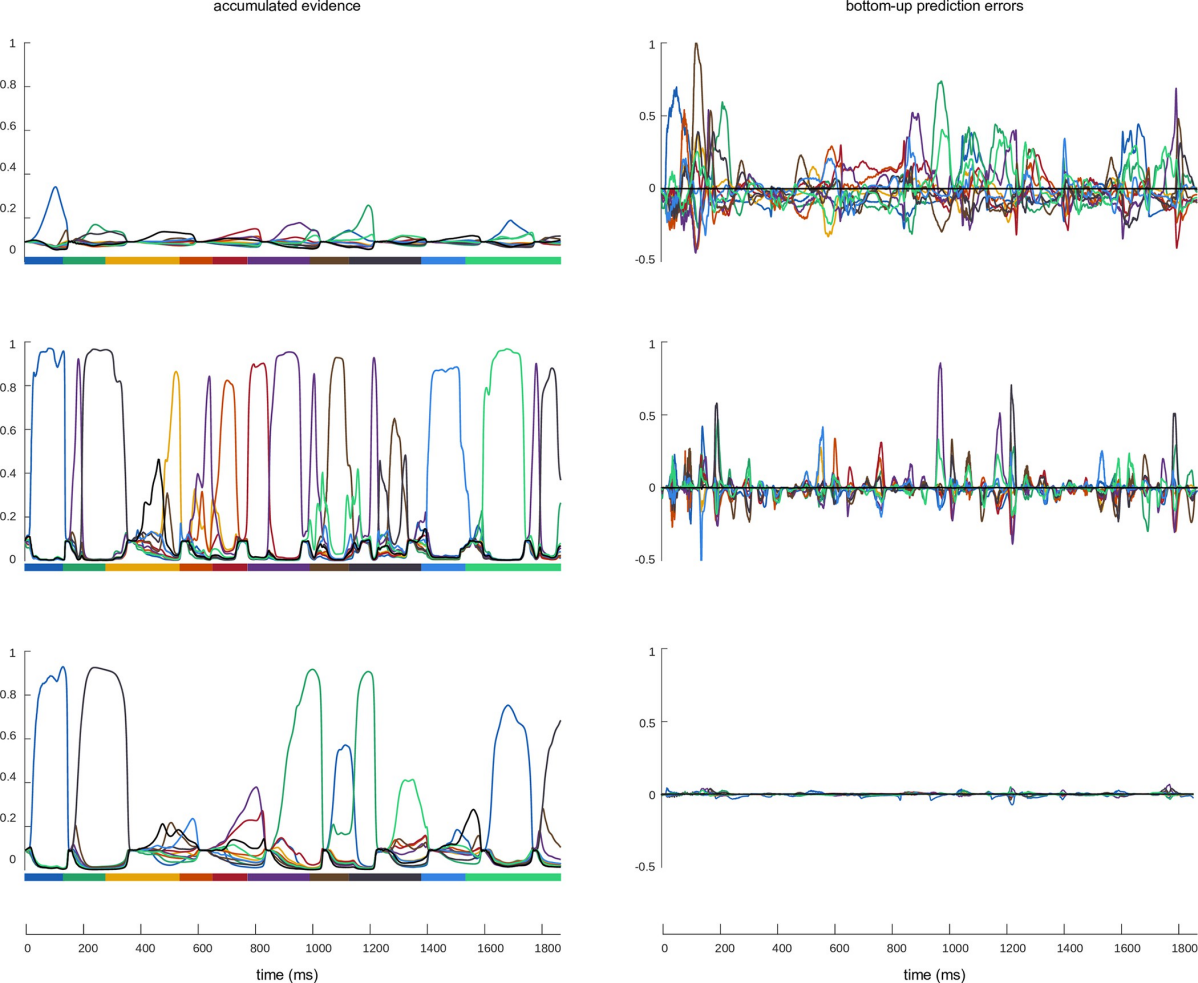
Effect of oscillating PEP on evidence accumulation. The left column represents accumulated evidence (softmax of syllable hidden states, colour coded, coloured dashes under each panel represent syllable sequence in the input sentence) and the right column represents bottom-up prediction errors about syllables (which carry the information from the input, colour coded). The rows indicate different conditions. Top: Precoss with fixed, very low, precisions; middle: Precoss-beta with oscillating precisions (20 Hz case); bottom: original Precoss (high precisions). The comparison between these variants illustrates that the oscillating PEP allows the model to integrate sensory information into the internal states more efficiently as evidenced by higher accumulated evidence.

The model encompasses two distinct functional groups operating in two distinct regimes: when the causal states of one group (syllable and/or gamma units) are in the low precision phase of the oscillation, they are both less strongly receptive to the internal expectations encoded by the hidden states and more strongly influenced by the bottom-up input carrying prediction errors from the periphery (Figs 5 and S6). As a result, each functional group is periodically in an optimal position to respond to bottom-up information without being constrained by internal expectations. And vice versa in the high-precision phase, where causal states are preferentially coupled to hidden states encoding internal expectations and more loosely to bottom-up input. The high-precision phase is therefore ideal to incorporate updates from the preceding low-precision phase into the internal hidden states. Thanks to oscillating PEPs, the model is rhythmically alternating between an information gathering and an information consolidation regime. The newly consolidated information leads to updated predictions, which in the next cycle are again compared with the input leading to updates in causal states, and to a new round of consolidation. That *Precoss-β* outperformed *Precoss* for almost all PEP frequencies indicates that rhythmic alternation of top-down and bottom-up streams during the inference process improves online syllable recognition. An important issue is therefore whether there is an optimal oscillating PEP rate in speech processing.

### Beta as an optimal range for rhythmic PEP

The different variants of *Precoss-β* were assessed based on three different metrics. One that assesses syllable accuracy and duration (Figs 2, 4A), one that quantifies the efficiency of the integration of bottom-up information about syllable identity (Fig 3) and another that additionally takes into account the uncertainty about syllable identity (Figs 4B and S7).

When judged on a metric assessing accuracy and duration (Fig 2), performance for all model variants (as assessed by syllable accuracy and duration) reaches a plateau rather than showing a peak frequency. The knee point of the plateau differed from variant to variant: 5 Hz for *Precoss-β-identity*, which roughly corresponds to the natural syllabic rhythm, and 20 Hz for *Precoss-β-timing*, a relatively sensible result given that gamma units are designed as a stable heteroclinic channel where activity within neighboring units can overlap in time.

For *Precoss-β-full*, with the knee point at 20 Hz, the performance attained was higher than for *Precoss-β-identity and Precoss-β-timing*, indicating an additive benefit of controlling PEP in both syllable and gamma units. This additive effect is larger when the modulation of syllable and gamma units is in anti-phase (Fig 4A), i.e. when one functional group is in a high precision state while the other is in a low one. In the anti-phase condition, only one functional group at a time (the one in the low-precision phase) can incorporate changes in the input, while the other incorporates information from the causal states into the dynamics. This alternation regime reduces the search space compared to the variant where the model tries to optimize syllable and gamma units simultaneously. However, this comes at a cost; if in addition to performance in terms of accuracy and duration, we also consider how clearly the syllables were detected, the advantage of the anti-phase alternation variant disappears for most modulation frequencies (S7 Fig). This could be due to the fact that although anti-phase alternation can reduce the search space by optimizing syllable and gamma units in a sequential/alternating way, there are always large unexplained bottom-up prediction errors in either syllable or gamma units, depending on which is in the low-precision state. In contrast, in other model variants, the low-precision phase occurs only for half of the sentence duration (*Precoss-β-identity*—PEP of syllable units only, *Precoss-β-timing*—PEP of gamma units only, and *Precoss-β-full-samephase*—simultaneously in both functional groups), suggesting that during the other half, when prediction errors are integrated into the hidden states, syllable and/or gamma are less perturbed by prediction errors. That is why, the entropy-weighted overlap metric would be less penalized in these model variants (S7 Fig and S20 Table).

Interestingly, for the full model (Fig 4), the beta range behaves differently from the other frequencies for both metrics. For the overlap metric (Fig 4A), which is based on a categorical decision about syllable identity, the difference between same-phase and antiphase is not significant in the beta region. For the entropy-weighted overlap metric, which takes into account how uncertain the syllable recognition was (S1 and S7 Figs) and corresponds to a probabilistic decision about the syllable identity in the input, the same-phase alternation variant is better than the anti-phase variant in the beta range. The latter is arguably the more neurophysiologically plausible scenario, as the probabilities of candidate syllables are passed up the speech hierarchy to form words based on the available context and grammar rules.

Theoretically, the appropriate rhythm to control precisions within early speech processing stages should be both slow enough to span across processing stages (and modules) and fast enough to achieve an optimal balance between input sensitivity and prediction updating. The beta range, intermediate between theta and gamma, is ideally suited for both purposes.

Although higher PEP frequencies might result in better syllable identification performance within a reduced hierarchy considered in our model (Figs 2 and S8), the beta range might be preferable within a larger hierarchy, as beta oscillations are considered to be a channel for long-range communication [42,43,54,55]. Beta oscillations that originate in higher levels of the cortical hierarchy could modulate precisions via a cascade running down from higher cognitive levels (semantics, syntax) to the lower cognitive levels (e.g. syllables) and sensory areas.

### Rhythmic PEP and precision theories

The beta rhythm has been linked to sensorimotor precisions before [56,57]. Sensorimotor beta activity reflects the integration of the sensory signal uncertainty with the uncertainty of the internal model about prediction errors in an adaptation task [48]. Here, we confirm the implication of the beta rhythm during inference and go further in showing 1) that the rhythmic modulation of precisions changes the relative weight of bottom-up vs. top-down information online, during the inference process, and 2) that this is beneficial in an eminently dynamic task such as online speech recognition. In other words, while precision (via e.g. synaptic gain) is important to assign uncertainty about the input throughout the hierarchy, there is an added benefit when it oscillates. Given bottom-up processes in the gamma and theta ranges, beta oscillations provide an optimal *timescale* to update precisions.

Although Precoss-β’s architecture is geared towards speech perception/syllable recognition, the neural timescales used are not specific to speech [44,45,58]. Oscillating precisions in the beta range may be beneficial for a wider range of cognitive operations than just syllable recognition, by facilitating top-down and bottom-up communication across processing steps and cortical regions.

In sum, the role of beta oscillations (or more generally the notion of oscillating precisions) is to rhythmically modulate the relative influence of top-down and bottom-up information flows on the fly during a multi-level inference process, here hierarchical speech processing. In this view, beta oscillations do not only act as an information channel [45,46], but as a gating mechanism of the top-down information flow.

### Rhythmic PEP and Predictive Routing

The rhythmic precision hypothesis is in line with studies suggesting rhythmic attentional sampling [59–62]. The *good* and *bad* phases associated with attentional sampling are conceptually similar to high and low precision states in the model. When bottom-up prediction errors have low precision, their contribution to the model dynamics decreases. This is similar to forming internal expectations while periodically scanning the sensory signal for something new or unexpected. Low precision phases provide windows of opportunity to detect new syllables in the input. In the absence of a new syllable, there is no substantial prediction error and the current syllable unit remains the most active one. Conversely, a new syllable triggers prediction errors which will, at the next increased precision phase, switch the corresponding syllable unit to its active form. The alternation of low and high precision states also fits with recent proposals linking cortical oscillations to the ability of neural networks to switch between attractors and therefore being able to efficiently sample from the space of available hypotheses [63].

This scenario works when there are already internal expectations formed about the sensory signal. For example, when subjects listened to short stories, beta activity built up as more context became available [25]. As the current model does not include higher hierarchical stages (word, phrase levels) it implicitly assumes that expectations are already formed and that there is ongoing beta activity. This assumption is sufficient to demonstrate that oscillating precisions can help online syllable recognition. However, in the brain, beta activity appears as bursts of transient activity when top-down predictions are possible. Bastos and colleagues (2020) introduced *predictive routing* as an implementation of hierarchical processing during visual perception [36]. Predictive routing assumes that alpha/beta bands prepare the pathways to process the predicted input by inhibiting bottom-up sensory information communicated at the gamma scale. Electrophysiological recordings showed enhanced alpha (8-14Hz) and beta (15-30Hz) activity for predictable stimuli, and gamma activity (40-90Hz) for unpredictable ones, especially in the lower layers of the hierarchy [36]. These results may also be explained by beta activity controlling precisions; when the stimulus is predictable and internal expectations are formed, beta activity originating from higher cortical areas modulates precisions throughout the whole hierarchy, explaining more alpha/beta power across the hierarchy for predictable signals. For unpredictable stimuli, there are no internal expectations and no need for an alternated contribution of top-down and bottom-up streams. In this case, the system takes in sensory information with more bottom-up activity communicated by gamma oscillations. The predictive routing framework can in our opinion comfortably accommodate the notion that beta oscillations control state precisions, and mediate the contribution of top-down and bottom-up information during the hierarchical (inferential) perception process.

By reflecting oscillating PEPs, beta activity may actually represent the top-down information rhythm. How such a functional theory could be implemented at the biophysical level remains to be established, but it is not incompatible with models of beta rhythm generation [54,64,65].

### Neurophysiological plausibility and comparison with other speech perception models

As in our original Precoss paper [49], we used a well-established model of the auditory periphery [50] as a basis for constructing the inputs to the model: a reduced auditory spectrogram, as well as its slow amplitude modulation. The model [50] captures some of the basic transformations that take place in the subcortical auditory system; including the transformation into an “auditory spectrogram”; a time-frequency representation of the sound, that takes into account the loss of temporal precision that happens in the brainstem. Although the auditory spectrogram in [50] uses 128 logarithmically spaced frequency bands, we reduced it to a 6-channel auditory spectrogram. This is sufficient to compare different neural architectures rather than recognition performance per se. Yildiz and colleagues (2013) used a similar approach in their speech recognition model [66]. Such a reduction is not unrealistic as it is known from the cochlear implant literature [67,68] that a 6-channel spectrogram contains enough information to decipher speech. The other input component, the slow amplitude modulation, is computed by convolving the auditory spectrogram with a spectrotemporal filter optimized for syllable boundary detection [17]. These inputs are then processed by two main modules: a theta module, and a spectro-temporal module that includes syllable and gamma units. This choice is based on the crucial role that theta and gamma oscillations play in speech perception [16,21,22], and their presence in auditory cortex [22,69,70]. Hyafil and colleagues [17] showed that coupled theta-gamma oscillations can successfully segment (theta oscillations) and decode (gamma activity) a continuous speech signal into syllable-like chunks. In the current study, instead of implementing theta and gamma with spiking neural networks as in Hyafil et al. (2015), we used a canonical theta neuron [71] to model the theta rhythm and a stable heteroclinic channel [72,73] operating at the gamma rate. The latter is particularly suitable for modelling sequential dynamics (such as the spectrotemporal pattern of a syllable) and can be obtained from neural mass models of membrane and action potentials (for details see [66,72–74]. This implementation of the neural oscillations captures the essential timescales and intended functions of these neural rhythms: segmentation and decoding/processing.

While Precoss-β, in contrast to most speech perception models [75–77], only covers the lower levels of the speech perception hierarchy and is simpler than contemporary ASR models [78,79], its main added feature is that it works on-line and potentially with low resources. Coupled with existing language models (e.g., GPT [80,81]), it could presumably reach high performance in on-line speech recognition.

Compared to existing speech perception models such as TRACE [75], Shortlist [76] and Shortlist B [77] its originality lies in that it combines hierarchical predictive processing [7,33,82] and neural oscillations [13,16,83], two theoretical frameworks playing a key role in speech perception. Although TRACE also implements a hierarchy of linguistic features (phonetic features, phonemes, words), where each level receives feedback from higher levels [75], this hierarchy is not based on the predictive coding/free energy principle [5,84].

Further, continuous signal segmentation [1,16] is also fundamentally different in Precoss and Precoss-β. Our point is that segmentation can be handled by coupled theta and gamma neural networks in a purely bottom-up fashion [17], but is further improved when top-down onset predictions are based on internal expectations about duration of speech segments based on higher level context [85]. Precoss implements a simpler version of theta oscillation-based syllable onset tracking and endogenous syllable duration estimation to decompose continuous speech into discrete syllable sequences [49]. This contrasts with the models mentioned above which use either discretized phonemes (Shortlist–[76]) or continuous phonemic features (TRACE–[75]).

Finally, our model is distinct from TEMPO, another model that uses nested neural oscillations. TEMPO uses a hierarchy of nested neural oscillations in the theta, beta and gamma range for syllable recognition [86]. Precoss-β, however, is a hierarchically structured generative model with both feedforward and feedback connections. While syllable recognition in TEMPO is organized by template matching at different timescales determined by the corresponding neural oscillation rhythm (theta tracks syllables, beta and gamma track dyads and phonemes, respectively), Precoss-β assigns different functions to different rhythms (theta for syllable tracking, beta controlling precisions and gamma providing processing windows), making it more neurophysiological plausible.

Although the current model is not intended to compare to automatic speech recognition models, Precoss-β could be used to improve them (e.g. [78,79]). The comparison is applicable to ASR models that use long-short-term memory (LSTM) [87,88] units with recurrent neural networks (RNN) [89], such as Deep Speech 2 [78], Listen Attend and Spell [79], to name a few. Comparison with these types of models is relevant because models using RNN generally analyze speech sounds in an incremental way (like Precoss/Precoss-β). LSTMs use forget gates to control the flow of information in and out of the memory units/cells, allowing the network to selectively retain or discard useful/required information. This mechanism allows LSTMs to process long-term relationships in the input signal (e.g., speech). Forget gates in LSTM are somewhat comparable to the oscillating PEP in Precoss-β, which change the influence of top-down vs. bottom-up information on the inference process. The main difference, however, is that forget gates selectively retain/discard information (e.g., based on input current, activity at previous time steps, weights, etc.), whereas oscillating PEP alternate the influence of top-down (bottom-up) information in a non-selective way whenever internal information is available, which is both more computationally advantageous and biologically plausible. Furthermore, while LSTMs are designed to process long-range dependencies in sequential data, oscillating PEP in the beta range coordinates information across a hierarchy of cortical levels. Our results suggest that LSTMs (and perhaps other ASR systems) may also benefit from the introduction of active, explicit oscillatory activity.

## Conclusion

This computational study suggests a new functional role of cortical oscillations in the specific context of hierarchical syllable recognition from natural sentences. First, we show that online syllable recognition benefits from oscillating precisions that alternate the contribution of top-down and bottom-up streams during the perceptual inference process. The performance gain is most tangible when functional groups responsible for different speech features alternatingly integrate bottom-up information and maintain internal expectations. The best performance (% recognized syllables, confidence and efficiency) is attained when the model controls precisions across functional groups in the 20–30 Hz range. Oscillating PEPs allow the model to reactively detect changes in the input, while maintaining internal expectations. These results entail a new mechanistic role for the beta range in speech processing, which might generalize to other cognitive functions relying on temporal information integration (e.g., spatial navigation). Oscillating precisions might represent a powerful strategy for the brain to swiftly transition from one high confidence hypothesis to another, and quickly sample its internal models. This is especially relevant for real world stimuli, which are never stationary, speech being a prime example. Although here we only considered the transition from continuous spectrotemporal patterns to discrete syllables, we propose that the same benefits would be obtained at all levels of the language network, up to the semantics and syntax levels, perhaps at other preferred frequencies. We also believe that our proposed implementation could be advantageous to produce low-resource artificial ASR and language models working on-line by allowing an incremental way of flexibly and dynamically combine internal expectations with the continuous and changing input that characterizes speech.

## Methods

### Speech input and syllabification

We have used the same set of 220 sentences from the TIMIT dataset [51] that we used in [49] for the simulations of the new model—Precoss-β. Briefly, for each sentence, a 6-channel reduced auditory spectrogram was calculated with a biologically plausible model of the auditory periphery [50]. Additionally, slow amplitude modulation of the sentence waveform was calculated following procedures described in Hyafil and colleagues [17,90].

Syllable boundaries in the input sentences were defined with the Tsylb2 [91] program based on the phonemic transcriptions provided in the TIMIT database [51]. The program estimates syllable boundaries based on English grammar rules, using phoneme annotations from TIMIT. Finally, syllable spectrotemporal patterns are calculated and stored in 6x8 matrices (6 frequency channels x 8 gamma units), where each row corresponds to the average value of the corresponding frequency bands within 8 binned temporal windows (assigned to specific gamma unit). For a detailed description of input construction and syllabification, please see the Methods section in [49].

### Generative model and *Precoss-β*

We use predictive coding to construct a model for parsing and recognizing syllables from natural English sentences. Inference is achieved by inverting a generative model. The generative model has two hierarchical levels, with the top level containing syllable and gamma units and the theta module. The latter signals syllable onset and rate information (S1 Text, Eqs 1–4) to the gamma units (S1 Text, Eqs 5–7). Finally, syllable units, which accumulate information about associated syllables, are modelled as perfect integrators (S1 Text, Eq 10). The bottom level features a Hopfield attractor that models the amplitude fluctuations of the frequency channels (S1 Text, Eqs 14–15). Finally, the causal states link the model levels and the model’s prediction about the input (S1 Text, Equs 11–13 and 17).

*Precoss-β* has the same hidden and causal states as in the original Precoss [49], but is defined with two additional hidden states at the top-level (full model equations are provided in the S1 Text). These represent the harmonic oscillator that controls the precision of syllable and/or gamma units:

$$\frac{dp_{1}}{dt}=k_{1}p_{2}+\varepsilon_{p_{1}}^{\left(2\right)}$$ (1)

$$\frac{dp_{2}}{dt}=-k_{1}p_{1}+\varepsilon_{p_{2}}^{\left(2\right)}$$ (2)

$$k_{1}=\frac{2\pi \psi }{1000}$$ (3)

Eqs 1 and 2 correspond to the oscillating precisions, Ψ in Eq 3 corresponds to the modulation frequency of prediction error precisions in Hz and 1000 is the sampling rate. We have tested each Precoss-β variant for different values of the modulation frequency Ψ ranging from 2 Hz up to 60 Hz. Table 1 contains precisions for new hidden states and oscillating causal states for each model variant.

**Table 1:** Precisions of syllable, gamma units and hidden states of the oscillating precisions. The left column represents stationary precisions for syllable and gamma units W_⍵_ and W_γ_ respectively, and for the new hidden states that generate oscillating precisions—W_p_. The right column represents the precision of causal states for each variant. Depending on the Precoss-β variant either syllable (*Precoss-β-identity*) or gamma (*Precoss-β-timing*) units have oscillating precision. Meanwhile, for variants Precoss-β-full (same/anti-phase), both syllable and gamma units have oscillating precisions, with the difference that for variant *Precoss-β-full-antiphase* they oscillate in opposite phases, while for *Precoss-β-full-samephase* in the same phase.

	hidden states	causal states
*Precoss-β-identity*	W_⍵_ = exp(3) W_γ_ = exp(5) W_p_ = exp(5)	V_⍵_ = exp(2.5+2p_2_) V_γ_ = exp(1.5)
*Precoss-β-timing*	W_⍵_ = exp(3) W_γ_ = exp(5) W_p_ = exp(5)	V_⍵_ = exp(5) V_γ_ = exp(1.5+4p_2_)
*Precoss-β-full-antiphase*	W_⍵_ = exp(3) W_γ_ = exp(5) W_p_ = exp(5)	V_⍵_ = exp(2.5+2p_2_) V_γ_ = exp(1.5-4p_2_)
*Precoss-β-full-samephase*	W_⍵_ = exp(3) W_γ_ = exp(5) W_p_ = exp(5)	V_⍵_ = exp(2.5+2p_2_) V_γ_ = exp(1.5+4p_2_)

The core difference between *Precoss* and *Precoss-β* is the inversion scheme used for inference: Dynamic Expectation Maximisation [92] for Precoss, and Generalized filtering [93] for *Precoss-β*. The latter features state-dependent precisions [94], which we use to actively modulate the precision of bottom-up prediction errors of syllable and/or gamma units.

For details about common aspects for *Precoss* and *Precoss-β*, we refer to [49].

### Overlap metric

The overlap metric was designed in order to assess model’s ability to accurately determine syllable identity and duration of the input sentence (S1 Fig). It measures the consistency between the recognized sequence of syllables and the sequence of syllables in the input sentence. This metric penalizes any discrepancies in either syllable identity or duration between the recognized and input syllables.


$$s_{j}^{\left(i\right)}(t)=\left\{ \begin{array}{l} 1,\;T_{j}^{\left(i)(start\right)}\leq t\leq T_{j}^{\left(i)(end\right)}\\ 0,\;otherwise \end{array}\right.$$ (4)

$$G^{\left(i\right)}=[0\ldots T_{\gamma }\ldots T^{\left(i)(end\right)}]$$ (5)

$$r_{j}^{\left(i\right)}(G_{k}\leq t\leq G_{k+1})=\left\{ \begin{array}{l} 1,\;if\;\mathrm{argmax}(M^{\left(i\right)})=j\\ 0,\;otherwise \end{array}\right.$$

$$M_{j}^{\left(i\right)}=\frac{1}{G_{k+1}-G_{k}}\sum_{\tau =G_{k}}^{G_{k+1}}\psi_{j}^{\left(i\right)}(\tau )$$ (6)

In the equations above $s_{j}^{\left(i\right)}(t)$ represents the syllable sequence in the input sentence $i(j=[1,N_{syl}^{\left(i\right)}]$), that contains $N_{syl}^{\left(i\right)}$ syllables. $T_{j}^{\left(i)(start\right)}$ and $T_{j}^{\left(i)(end\right)}$ represent the start and end timepoints of syllable *j* in the sentence *i*. *G*^(*i*)^ represents syllable boundaries based the internal, gamma-based time markers (*T*_*γ*_, all time points where that last gamma unit (Eq 8 in S1 Text) *y*_8_(*T*_*γ*_) is a local maximum with the amplitude of at least 0.6). $T^{\left(i)(end\right)}=T_{N_{syl}^{\left(i\right)}}^{\left(i)(end\right)}$ is the endpoint/duration of sentence *i*. It is important to note, that in principle it could happen that there are more/less gamma-based syllable segments (*K*^(*i*)^) than the number of syllables in the sentence $N_{syl}^{\left(i\right)}$. For each gamma-based segment, we look at which syllable has the highest average activation (Eq 6, where *r*^(*i*)^(*t*) is the recognized syllable sequence and $\psi_{j}^{\left(i\right)}$ is the *j* -th component of SoftMax of syllable hidden states (Eq 12 without the noise term in S1 Text)). Overlap metric for sentence *i* is defined as a ratio of dot product between recognized (*r*^(*i*)^(*t*)) and input syllable sequences *s*^(*i*)^(*t*) divided to the duration of the sentence *T*^(*i*)(*end*)^, Eq 7.


$${P_{overlap}}^{\left(i\right)}=\frac{1}{T^{\left(i)(end\right)}}\sum_{t=0}^{T^{\left(i)(end\right)}}\sum_{j=1}^{N_{syl}^{\left(i\right)}}s_{j}^{\left(i\right)}(t)r_{j}^{\left(i\right)}(t)$$ (7)

### Entropy-weighted overlap metric

The overlap-metric described above makes a categorical decision about the identity of a syllable (Eq 4), however it does not take into account the uncertainty associated with the activated syllable. Entropy-weighted overlap metric is designed to address this issue. First, for each sentence, *i* we calculate the entropy associated with the SoftMax of syllable hidden states *ψ*^(*i*)^

$$E^{\left(i\right)}(t)=\frac{1}{\mathrm{lg}(N_{syll}^{\left(i\right)})}\sum_{j=1}^{N_{syll}^{\left(i\right)}}\psi_{j}^{\left(i\right)}(t)\mathrm{lg}\psi_{j}^{\left(i\right)}(t)$$ (8)

Were we also normalized with the maximum possible entropy of sentence *i* that has $N_{syll}^{\left(i\right)}$ syllables. The *E*^(*i*)^(*t*) is low when the model selected only one(few) candidate(s) for the input syllable (this what we want to award in this new metric), and high when there are many candidates (this we want to penalize). Therefore, the entropy-weighted overlap metric for each sentence *i* was defined as follows:

$${P_{ent-overlap}}^{\left(i\right)}=\frac{1}{T^{\left(i)(end\right)}}\sum_{t=0}^{T^{\left(i)(end\right)}}(1-E^{\left(i\right)}(t))\sum_{j=1}^{N_{syl}^{\left(i\right)}}s_{j}^{\left(i\right)}(t)r_{j}^{\left(i\right)}(t)$$ (9)

### Longest common-subsequence

The longest common-subsequence (lcs) metric for each sentence is based on the longest subsequence that is present in both sequences, where the subsequence is obtained by deleting items without any additions or changes in order [95]. For example, if we compare the sequence a = [8, 1, 3, 2, 4, 5, 5, 7] with the sequence b = [1, 2, 3, 4, 5, 6, 7, 8], we will find that the LCS between a and b is c = [1, 2, 4, 5, 7]. In our case we calculate lcs between the syllable sequence in the input $s_{input}^{\left(i\right)}(j)=j$ (where $j=1\ldots N_{syl}^{\left(i\right)}$) and $s_{rec}^{\left(i\right)}(k)=r^{\left(i)(-1\right)}(G_{k})$ (where $k=1\ldots K^{\left(i\right)},r^{\left(i)(-1\right)}(t)$ is the inverse of the function in Eq 6 and returns the index ($j=1\ldots N_{syl}^{\left(i\right)}$) of the recognized syllable). Model performance based on the lcs metric for sentence *i* is, therefore, defined as:

$$P_{lcs}^{\left(i\right)}=\frac{L(s_{input}^{\left(i\right)},s_{rec}^{\left(i\right)})}{N_{syl}^{\left(i\right)}}$$ (10)

Where $L(s_{input}^{\left(i\right)},s_{rec}^{\left(i\right)})$ donates to the length of the longest common subsequence between $s_{input}^{\left(i\right)}$ and $s_{rec}^{\left(i\right)}$. Importantly, contrary to the overlap and entropy-weighted overlap metric, the lcs metric depends only on the identity (index) of the recognized syllables and is not sensitive to how well the model was able to infer syllable durations.

### Sensory information integration efficacy

As mentioned above, the oscillating PEP also affects how well the model is able to integrate the sensory information (bottom-up prediction error). To quantify this effect, we tracked when each syllable’s hidden state changed in the same direction as the corresponding component of the bottom-up prediction error. In other words, a positive bottom-up prediction error means that there is information about the corresponding syllable in the input, and the model should integrate this information into the corresponding syllable hidden state. If the model is successful, the value of the corresponding syllable hidden state would change positively (the derivative would be positive). Similarly, a negative prediction error means that the corresponding syllable hidden state does not represent the syllable in the input, so its activity should decrease (negative derivative) if the model is successful in interpreting this information. Mathematically, this can be described by calculating the dot product (component wise) between the positive (negative) derivative of the hidden syllable states and the corresponding positive (negative) prediction error over the sentence duration (Eq 4).


$$r_{j}^{i}=\frac{1}{T}\sum_{t=1}^{T}h(t)[z_{i}^{+}(t){\left(\frac{d\omega_{i}(t)}{dt}\right)}^{+}+z_{i}^{-}(t){\left(\frac{d\omega_{i}(t)}{dt}\right)}^{-}]$$ (11)

$$R_{j}=\frac{1}{N_{syl}}\sum_{i=1}^{N_{syl}}r_{j}^{i}$$ (12)

First, for each syllable *i* in sentence *j* we calculate the mean the product of positive evidence in favor of syllable *i* $z_{i}^{+(-)}(t)$ and derivative of its syllable hidden state ${\left(\frac{d\omega_{i}(t)}{dt}\right)}^{+(-)}$, where the summation is across time but excluding periods of active resetting of syllable units that happens when the gamma network signals the end of a syllable (for details see [49]). This is achieved by defining *h*(*t*), which is one outside active resetting periods and 0 during active resetting periods.

We also average root sum square (*R*_*j*_) across all syllables *N*_*syl*_ of sentence *j* (Eq 5). Each dot in Fig 3 indicates the value *R*_*j*_ for each sentence (j) for corresponding PEP frequency.

### Bayesian information criterion

Compared to the original Precoss model, the new Precoss-β model is more complex as it includes two additional variables responsible for the generation of PEP oscillations. To account for the additional complexity of the model (19 parameters (Precoss-β) vs. 17 parameters (Precoss)), we calculated the BIC values based on the probabilities assigned by the model to the syllables in the input. The syllable hidden states correspond to the evidence accumulated about each syllable during the inference process, while the SoftMax of the syllable hidden states represents the probabilities assigned by the model about each syllable in the input sentence. Therefore, the log-likelihood for a categorical distribution with N possible outcomes (N is the number of syllables in the input sentence *s*^(*i*)^ for model m) would be:

$$\begin{array}{c} \log\;p({s_{j}}^{\left(i\right)}|m)=\sum_{t=1}^{T}\log(s_{j}^{\left(i\right)}(t)\psi_{j}^{\left(i\right)}(t))\\ \log\;p(s^{\left(i\right)}|m)=\sum_{j=1}^{N_{syl}^{\left(i\right)}}\frac{1}{d_{j}^{\left(i\right)}}\log\;p({s_{j}}^{\left(i\right)}|m) \end{array}$$ (13)

Where, in the first equation, for each sentence *i*, the $s_{j}^{\left(i\right)}(t)$ and $\psi_{j}^{\left(i\right)}(t)$ correspond to the syllable in the input (Eq 4) and model assigned probability to that syllable (the corresponding component of the SoftMax of syllable hidden states) at time t, respectively. Finally, to control for syllables with different durations, we divide the log likelihood of each syllable by its duration $d_{j}^{\left(i\right)}$ before summing them to get a single value for a sentence.

The BIC value for each model variant was calculated by:

$$BIC(m)=\sum_{i=1}^{N_{sent}}log\;p(s^{\left(i\right)}(t)|m)-0.5N_{sent}logN_{p}(m)$$ (14)

Where *N*_*sent*_ and *N*_*p*_(*m*) N_p_(m) stand for the number of sentences used in simulations and number of parameters in model m, respectively.

The BIC values for all Precoss-β variants for all PEP frequencies are shown in the S29 Table.

### Statistical analysis

The model performance was evaluated based on the overlap metric (S1 Fig) that provides a single value for each sentence assessing the model’s ability to infer syllable identity and duration for each sentence. Simulations were performed on the same set of 220 sentences for each model variant and each frequency of modulation of prediction error precisions.

To compare the performance of *Precoss* vs *Precoss-β* we performed a Wilcoxon signed-rank test for each PEP frequency. To control for multiple comparisons the alpha = 0.05 was adjusted with the Bonferroni procedure. Each test was considered statistically significant if the p-value was less than 0.05/8 (dominator corresponds to the number of comparisons—the number of tested frequencies). The same method was used for the same-phase vs. anti-phase conditions (presented in Fig 4). Results are presented in S1–S3, S13–S15, S25–S27 Tables (for comparisons based on the overlap, entropy-weighted overlap and lcs metrics, respectively) for *Precoss-β* variants, and S9 Table for anti-phase vs same-phase comparisons. In all tables the first column indicates which frequency is tested, the second column the associated signed-rank, and the third column the corresponding z-statistics. The last column represents the corresponding p-value.

We have also compared Precoss-β variants with N-way ANOVA (anovan function in RMatlab2020b). We have looked at interaction between two factors (model type–PEP of which units is controlled) and frequency of PEP set as continuous predictor.

For each *Precoss-β* variant, the effect of the modulation frequency was evaluated with a Friedman test, followed by multiple comparisons controlled by Bonferroni correction. S4-7 (for overlap metric), S10-S12 (integration efficiency), S16-S19 (for entropy-weighted overlap metric) and S21–S24 Tables (lcs) report results of pairwise comparisons, where the first two columns indicate which modulation frequencies of precisions are being compared. The fourth column indicates the difference in the mean signed-rank for the corresponding pair, whereas the third and fifth columns indicate lower and upper bound of 95% confidence interval, correspondingly. Lastly, the sixth column represents Bonferroni corrected p-values. Pairwise comparisons are considered statistically significant if the corrected p-value < 0.05.

All statistical tests were performed using built-in Matlab functions. Sentences (32, 64, 70, 77, 131) did not converge for *Precoss-β-full-samephase*, thus were excluded from same-phase vs anti-phase comparisons (Fig 4).

## Supporting information

S1 Text Includes the detailed mathematical description of the original Precoss model [49]. (DOCX)

S1 Fig Performance metric overlap based on the dynamics of syllable and gamma units. The top two panels represent the dynamics of the gamma and syllable hidden states during inference for an example sentence. For each subplot, colored lines were used to represent different gamma and syllable units. The gamma unit with a thick blue line corresponds to the first gamma unit, whose peak (amplitude more than 0.6) is used as a marker to indicate windows for identifying the “winner” syllable unit. For the latter, we look for the syllable unit with the highest average activation within a gamma window (time interval between two consecutive gamma 1 peaks). The sequence of the recognized syllables is shown in the 3rd panel (colored solid lines), whereas the dashed line indicates the entropy associated with the softmax of syllable hidden states (top panel). The sequence and duration of the syllables in the input are shown in the 4th subpanel. The model performance (the overlap metric) is evaluated with the sum of the dot-product (bottom subpanel) of recognized and input syllable sequences (subpanels 3 and 4) divided by the duration of the input sentence. The higher/closer to 1, the better the model is able to infer identity and duration of syllables in the input sentence. The overlap metric that also incorporates (Fig 4B) the entropy is calculated based on the sum of the dot product of recognized syllable sequence (solid lines on the 3^rd^ panel), 1-entropy (the dashed line on the 3^rd^ panel) and syllable sequence in the input (4^th^ panel).
(TIF)

S2 Fig *Precoss-β-identity* performance based on the overlap metric. Simulation results on 220 sentences. Performance is evaluated based on the overlap between the recognized syllable sequence and the sequence of syllables in the input sentence (for details, see S1 Fig). We compare the performance of *Precoss-β* for different frequency values of PEP. For all frequencies, performance is better than that of *Precoss* with stationary precisions (S1 Table). Friedman test (χ2 = 24.77, p = 0.0008) indicated an effect of PEP frequency on model performance. Post-hoc pairwise comparisons (Bonferroni-corrected, S4 Table), indicated that performance of *Precoss-β* increased with frequency up to 5 Hz and reached a plateau (there is no statistically significant difference in the model’s performance for frequencies higher or equal to 5 Hz). Each point on the scatter plot represents the model performance in each sentence for the corresponding PEP frequency. The central-red mark of the box plots indicates the median, whereas bottom and top edges represent 25th and 75th percentiles. Red crosses indicate outliers, whereas whiskers extend to the highest and lowest overlap values that are not considered outliers. The blue line at the top represents comparisons of *Precoss-β* with *Precoss*, while triangular grey lines indicate comparisons within *Precoss-β* for different PEP frequencies. Arrows on these lines indicate significant differences, while the direction of the arrows indicates the sign of the effect.
(TIF)

S3 Fig *Precoss-β-timing* performance based on the overlap metric. Simulation results on 220 sentences are presented in the figure. Performance is evaluated based on the overlap between the recognized and input syllable sequences (for details, see S1 Fig). *Precoss-β* outperforms *Precoss* for PEP frequencies higher or equal to 10 Hz, whereas for smaller frequencies the performance is worse (S2 Table). Friedman test (χ2 = 125.4, p = 5.727e-24) indicated an effect of PEP frequency on model performance. Post-hoc, multiple comparisons tests (corrected with Bonferroni procedure, S5 Table) indicated that *Precoss-β* performance increases with frequency and reaches a plateau at around 20 Hz. Each point on the scatter plot represents the value for each sentence for the corresponding PEP frequency. The central-red mark of the box plots corresponds to the median, whereas bottom and top edges represent 25th and 75th percentiles, respectively. Red crosses indicate outliers, whereas whiskers extend to the highest and lowest performance values that are not considered outliers. The blue line at the top represents comparisons of *Precoss-β* with *Precoss*, while triangular grey lines indicate comparisons between different PEP frequencies within *Precoss-β*. Arrows on these lines indicate significant differences, while the direction of the arrows indicates the sign of the effect.
(TIF)

S4 Fig *Precoss-β-full*-antiphase performance based on overlap metric. Simulation results on 220 sentences are presented in the figure. Performance is evaluated based on the overlap duration between the recognized syllable sequence and the sequence of syllables in the input sentence (for details, see S1 Fig). For this condition performance of *Precoss-β* is better than the performance of Precoss, with stationary precisions for all frequency values of the precision units (S3 Table). Friedman test (χ2 = 128.41.86, p = 1.351e-24) confirmed that the frequency of PEP affects model performance. Post-hoc, Bonferroni corrected pairwise comparisons indicated that the model performance increases with the frequency and reaches a plateau at 20 Hz (there are no statistically significant differences in performance for higher PEP frequencies, S6 Table). The central-red mark of the box plots corresponds to the median, whereas bottom and top edges represent 25th and 75th percentiles, respectively. Red crosses indicate outliers, whereas whiskers extend to the highest and lowest model performance values that are not considered outliers. The blue line at the top represents comparisons of *Precoss-β* with *Precoss*, while triangular grey lines indicate comparisons within *Precoss-β* for different PEP frequencies. Arrows on these lines indicate significant differences, while the direction of the arrows indicates the sign of the effect.
(TIF)

S5 Fig *Precoss-β-full*-samephase performance based on overlap metric. Simulation results on 220 sentences are presented in the figure. Performance is evaluated based on the overlap duration between the recognized syllable sequence and the sequence of syllables in the input sentence (for details, see S1 Fig). For this condition performance of *Precoss-β* is better than the performance of Precoss, with stationary precisions for all frequency values of the precision units (S3 Table). Friedman test (χ2 = 94.94.86, p = 1.192e-17) confirmed that the frequency of PEP affects model performance. Post-hoc, Bonferroni corrected pairwise comparisons indicated that the model performance increases with the frequency and reaches a plateau at 20 Hz (there are no statistically significant differences in performance for higher PEP frequencies, S6 Table). The central-red mark of the box plots corresponds to the median, whereas bottom and top edges represent 25th and 75th percentiles, respectively. Red crosses indicate outliers, whereas whiskers extend to the highest and lowest model performance values that are not considered outliers. The blue line at the top represents comparisons of *Precoss-β* with *Precoss*, while triangular grey lines indicate comparisons within *Precoss-β* for different PEP frequencies. Arrows on these lines indicate significant differences, while the direction of the arrows indicates the sign of the effect.
(TIF)

S6 Fig *Precoss-β* dynamics of syllable units for different PEP frequencies. Each panel in the figure represents the effect of PEP on the dynamics of syllable recognition for different PEP frequencies. Each panel contains 3 plots. The top one represents the softmax of syllable hidden states (colour coded for different syllables in the input sentence), with the dashed line representing the entropy associated with the accumulated evidence. The middle plot shows the syllable hidden states, with the horizontal the bars representing the syllable sequence (identity and duration) in the input sentence. The bottom plot represents the bottom-up prediction errors for the syllable units, with the dashed line corresponding to the oscillation controlling the precision of the prediction errors. This comparison illustrates that during low PEP frequencies low/high precision phase spans often extend over several syllables in the input. This means that for many syllables the model is not able to integrate and accumulate sensory information. In case of higher PEP frequencies there is "always" unexplained prediction errors, that results in more noisy dynamics of syllable causal states.
(TIF)

S7 Fig Model performance based on the entropy weighted overlap metric. The figure illustrates the model performance based on the entropy weighted overlap metric (S1 Fig). The graph shows the mean performance and 95% confidence interval after bootstrapping for different Precoss-beta variants (color coded) and Precoss with fixed precision (the blue band). Arrows at the top indicate significant differences within model comparisons for different PEP frequencies. The direction of an arrow indicates the direction of the effect. Similarly, the blue line and the arrows on it show the comparisons for each Precoss-beta variant versus Precoss, with the direction of the arrows indicating that Precoss-beta with oscillating precisions outperforms Precoss for all variants and PEP frequencies.
(TIF)

S8 Fig The longest common subsequence between recognized and input syllable sequences. The figure illustrates the evaluation of the model variants based on the longest-common-sub-sequence (lcs). For each sentence, the lcs between the recognized syllable sequence and the syllable sequence in the input sentence is retrieved. The length of the lcs is divided by the number of syllables in the input sentence, giving the percentage on which this figure is based. Thus, for each model variant (color coded) and for each PEP frequency, we show the mean lcs (in %) and the 95% confidence interval. The arrows (color coded) represent the statistically significant differences for within-model comparisons for different PEP frequency values. The direction of the arrows represents the direction of the effect (pointing to the left would mean that the frequency on the left has a statistically lower lcs value than the frequency on the right). Similarly, the blue line and the arrows on it represent the comparison between Precoss and Precoss-beta variants, where the arrows pointing downwards indicate that the corresponding Precoss-beta variant (color coded) and frequency has significantly better performance.
(TIF)

S1 Table *Precoss* vs. *Precoss-β-identity*. (XLSX)

S2 Table *Precoss* vs. *Precoss-β-timing*. (XLSX)

S3 Table *Precoss* vs. *Precoss-β-full-antiphase*. (XLSX)

S4 Table Multiple comparison table for Precoss-β-identity, overlap metric. (XLSX)

S5 Table Multiple comparison table for Precoss-β-timing, overlap metric. (XLSX)

S6 Table Multiple comparison table for Precoss-β-full-antiphase, overlap metric. (XLSX)

S7 Table Multiple comparison table for Precoss-β-identity-samephase, overlap metric. (XLSX)

S8 Table Comparison between model variants–overlap metric. 2-way ANOVA (model variant (discrete factor) and PEP frequency (continuous factor)) was performed to analyze Precoss-β variant on performance based on the overlap metric. Simple main effect analysis showed that model variant has statistically significant effect on the model performance (F = 15.92, p = 0). (XLSX)

S9 Table Effect of the oscillating PEP phase on model performance. Related to Fig 5. The performance difference (same-phase minus anti-phase) is considered statistically significant if the p < 0.05/8 (corrected for multiple comparisons with Bonferroni procedure). Sentences 32, 64, 70, 77, 131 were removed from analysis, as the model fell into singularities, which resulted in NAN values for the entropy calculations. The first line for each comparison corresponds to the overlap metric, where the significant differences are highlighted with the light gray shade. The second raw corresponds to the overlap metric that also incorporates entropy, here the significant differences are highlighted with the light blue shade.
(XLSX)

S10 Table Multiple comparison table for Precoss-β-identity—(integration efficiency). Results of Friedman test (χ^2^ = 28.55, p = 0.0002) and followed within PEP-frequency multiple comparisons (Bonferroni corrected) are presented.
(XLSX)

S11 Table Multiple comparison table for Precoss-β-timing—(integration efficiency). Results of Friedman test (χ^2^ = 605.71, p = 1.434e-126) and followed within PEP-frequency multiple comparisons (Bonferroni corrected) are presented. Sentence N182 was removed for this result, as model did not converge.
(XLSX)

S12 Table Multiple comparison table for Precoss-β-full—(integration efficiency). Results of Friedman test (χ^2^ = 269.85, p = 1.635e-54) and followed within PEP-frequency multiple comparisons (Bonferroni corrected) are presented.
(XLSX)

S13 Table *Precoss* vs. *Precoss-β-identity*, entropy-weighted overlap metric. (XLSX)

S14 Table *Precoss* vs. *Precoss-β-timing*, entropy-weighted overlap metric. (XLSX)

S15 Table *Precoss* vs. *Precoss-β-full*, entropy-weighted overlap metric. (XLSX)

S16 Table Multiple comparison table for Precoss-β-identity—(entropy weighted overlap metric). Results of Friedman test (χ^2^ = 53.04, p = 3.64e-9) and followed within PEP-frequency multiple comparisons (Bonferroni corrected) are presented.
(XLSX)

S17 Table Multiple comparison table for Precoss-β-timing—(entropy weighted overlap metric). Results of Friedman test (χ^2^ = 114.53, p = 1.05288e-21) and followed within PEP-frequency multiple comparisons (Bonferroni corrected) are presented.
(XLSX)

S18 Table Multiple comparison table for Precoss-β-full-antiphase—(entropy weighted overlap metric). Results of Friedman test (χ^2^ = 152.91, p = 9.918e-30) and followed within PEP-frequency multiple comparisons (Bonferroni corrected) are presented.
(XLSX)

S19 Table Multiple comparison table for Precoss-β-full-samephase—(entropy weighted overlap metric). Results of Friedman test (χ^2^ = 123.8, p = 1.236e-23) and followed within PEP-frequency multiple comparisons (Bonferroni corrected) are presented.
(XLSX)

S20 Table Comparison between model variants–entropy weighted overlap metric. 2-way ANOVA (model variant (discrete factor) and PEP frequency (continuous factor)) was performed to analyze Precoss-β variant on performance based on the entropy weighted overlap metric. Simple main effect analysis showed that model variant has statistically significant effect on model performance (F = 5.4, p = 0.001)
(XLSX)

S21 Table Multiple comparison table for Precoss-β-identity—(lcs metric). Results of Friedman test (χ^2^ = 47.42, p = 4.627e-8) and followed within PEP-frequency multiple comparisons (Bonferroni corrected) are presented.
(XLSX)

S22 Table Multiple comparison table for Precoss-β-timing, lcs metric. Results of Friedman test (χ^2^ = 125.85, p = 4.62e-24) and followed within PEP-frequency multiple comparisons (Bonferroni corrected) are presented.
(XLSX)

S23 Table Multiple comparison table for Precoss-β-full-antiphase, lcs metric. Results of Friedman test (χ^2^ = 159.29, p = 4.51e-31) and followed within PEP-frequency multiple comparisons (Bonferroni corrected) are presented.
(XLSX)

S24 Table Multiple comparison table for Precoss-β-full-samephase, lcs metric. Results of Friedman test (χ^2^ = 109.56, p = 1.13e-20) and followed within PEP-frequency multiple comparisons (Bonferroni corrected) are presented.
(XLSX)

S25 Table *Precoss* vs. *Precoss-β-identity*, lcs metric. (XLSX)

S26 Table *Precoss* vs. *Precoss-β-timing*, lcs metric. (XLSX)

S27 Table *Precoss* vs. *Precoss-β-full*, lcs-metric. (XLSX)

S28 Table Comparison between model variants–lcs metric. 2-way ANOVA (model variant (discrete factor) and PEP frequency (continuous factor)) was performed to analyze Precoss-β variant on performance based on the lcs metric. Simple main effect analysis showed that model variant has statistically significant effect on the model performance (F = 20.87, p = 0).
(XLSX)

S29 Table Bayesian Information Criterion. The highlighted cells correspond to the Precoss-β variants and PEP frequencies where the BIC values were higher than the BIC value of Precoss (-10057).
(XLSX)
