# Supplementary material for: Rhythmic modulation of prediction errors: A top-down gating role for the beta-range in speech processing
Source: PLoS Comput Biol. 2023 Nov 7;19(11):e1011595. doi: 10.1371/journal.pcbi.1011595 (PMC10655987; doi:10.1371/journal.pcbi.1011595)
Supplement: S1 Text — (DOCX) [file pcbi.1011595.s001.docx]

# Supplementary Text S1

## Input construction and syllable spectrotemporal patterns

We used 220 (containing 2888 syllables) English sentences from the TIMIT database [1] for our simulations, which were transformed into a time-frequency decomposition (auditory spectrogram) and slow amplitude modulation. We employed the early subpart of a biologically inspired model of the auditory periphery [2] to transform the audio signal into 128 frequency channels, which were then normalized (to [0 1] interval) and reduced to 6 channels. The slow amplitude modulation is calculated according to the procedures described in Hyafil et al. (2015) [3], which involved transforming the auditory spectrogram into 32 channels and convolving with a filter optimized to signal syllable boundaries. Overall, each sentence was represented by 7 input channels, the slow amplitude modulation (*S*(t)) and 6 frequency bands (*F_f_*(t); *f* = 1, … 6).

In addition, for each syllable (*ω*) of the input sentence, we calculated the corresponding spectrotemporal patterns ST*_fγω_* (*f* = 1, … 6, number of frequency channels and *γ* = 1, … 8, number of gamma units) by dividing the corresponding auditory spectrogram (of one syllable) into 8 equal duration time bins and averaging the values of each of the 6 frequency channels. Thus, for each syllable, the spectrotemporal patterns are matrices with 6 rows representing frequency channel and 8 columns representing temporal order (but not duration). These spectrotemporal patterns were stored (after non-linear transformation) in the model’s memory, and were used during the inference process to link the activity of individual syllable units to a spectrotemporal , deployed in time by the gamma units. During the simulation of each sentence, only the spectrotemporal patterns of the syllables of that sentence were stored in the model's memory. Further details can be found in the Methods section of the original paper [4].

## Mathematical description of Precoss

Below we present the equations of the generative model of the original Precoss model [4] that are shared with the new Precoss-β model. The new model has additional hidden states in the top level that generate the rhythm for the oscillatory prediction error precisions, that is presented in the Methods section. The equations below are the bare mathematical description of the shared modules, for more details please refer to the original paper.

### Top level

The model tracks the slow amplitude modulation in the input with a perfect integrator:

$$\frac{dA}{dt}=0+\varepsilon_{\text{A}}^{(2)}$$

We modify the tracked amplitude and use it as an input to the canonical theta model [5].

$$R=0,25+0,21A$$

The first parameter on the right-hand side is chosen so that theta frequency is around 5 Hz whenever *A* is 0. The coefficient for *A* ensures that the range of the resulting rhythm is within the biological theta range (3-8 Hz, tested on the first 10 sentences of the dataset).

The following pair of the equations corresponds to the theta oscillator in the theta module.

$$\begin{matrix} \frac{dq_{1}}{dt}=-kq_{2}\left(1+R+q_{1}\left(R-1\right)\right)+\varepsilon_{\text{q}_{1}}^{\left(2\right)} \\ \frac{dq_{2}}{dt}=kq_{1}\left(1+R+q_{1}\left(R-1\right)\right)+\varepsilon_{\text{q}_{2}}^{\left(2\right)} \\ k=\frac{2\pi\Omega}{1000} \end{matrix}$$

Where 1000 is the sampling rate and *Ω* = 5 Hz is the frequency of theta in the absence of input (*A* = 0). The quantity within brackets of the right-hand side of Equation 3 stands for the normalized instantaneous rate of the theta oscillation (instantaneous rate = *k∙s*_θ_).

$$s_{\text{θ}}=1+R+q_{1}\left(R-1\right)$$

The value *s*_θ_ to set the preferred rate of the gamma units (Equation 7).

Gamma units are modelled as a stable heteroclinic channel, which results in their sequential activation [6] (for details see [7,8]). The duration of the gamma sequence depends on the hidden variable *s* (Equation 7); which sets the rate of the gamma sequence through *κ_2_* in Equation 5.

The mathematical equations are adapted from Yildiz et al. (2013) [7].

$$\frac{dz}{dt}=\kappa_{2}(s)[-\lambda z-\rho S(z)+1]-\beta(z-z_{0})T_{\text{γ}}+\varepsilon_{\text{z}}^{(2)}$$

$$\frac{dy_{i}}{dt}=e^{z_{i}}-y_{i}\sum_{j=1}^{N_{\gamma}} e^{z_{j}}-\beta(y_{i}-y_{i,0})T_{\text{γ}}+\varepsilon_{\text{y}_{i}}^{(2)}$$

$$\frac{ds}{dt}=s_{\text{θ}}-s+\varepsilon_{\text{s}}^{\left(2\right)}$$

Where

- *i* takes values from 1 to *N*_γ_ = 8 and is the index of gamma units.
- the vector z encodes the amplitude fluctuations of the gamma units and the vector *y* is the amplitude of the gamma units scaled to the [0, 1] interval.
- z_0_ and y_0_ represent the reset values of z and y, corresponding to the state when the first gamma unit is active (the start of the gamma sequence)
- *T*_γ_ stands for the trigger that gamma units receive from the theta module and corresponds to a predefined phase of theta rhythm.
- *β* = 0.5 is a scaling factor for theta triggers
- *S*(z) = 1/(1+e*^-^*^z^) is applied component-wise.
- ρ*_ij_* ≥ 0 is the connectivity matrix, determining the inhibition strength from unit *j* to *i*. Its values are:

$$\rho_{ij}=\left\{\begin{matrix} 0 i=j \\ 1.5 j=i+1 \\ 0.5 j=i-1 \\ 1 otherwise \end{matrix}\right.$$

To ensure that the time constant κ_2_ stays positive we used the following transformation:

$$\kappa_{2}(s)=0.2625e^{\left(s-1\right)}$$

Finally, syllable units, that accumulate the evidence from the input associated with corresponding syllables, are modelled with the following equations.

$$\frac{d\text{ω}}{dt}=-(\text{ω}-\text{ω}_{0})T_{\text{ω}}+\varepsilon_{\text{ω}}^{(2)}$$

where omega is a vector with as many components as syllables in the sentence (plus a unit that encodes “silence” – all entries are 0). *T*_ω_ corresponds to the last gamma unit, signalling the end of the gamma sequence and, thus, the end of the generated spectrotemporal pattern of a syllable.

The causal states of the second level pass information to the bottom level:

$$\nu_{\text{γ}}^{(1)}=y+\eta_{\text{γ}}^{(2)}$$

$$\nu_{\text{ω}}^{(1)}=\frac{e^{-\omega}}{\sum e^{-\omega}}+\eta_{\text{ω}}^{(2)}$$

$$\nu_{\text{A}}^{(1)}=A+\eta_{\text{A}}^{(2)}$$

Where we also used the softmax function to scale the activity of the syllable units (Equation 18).

### Bottom level

The bottom level contains variables related to the amplitude fluctuations of the frequency channels as well as the slow amplitude modulation.

The amplitude fluctuations of the frequency channels are modelled with a Hopfield attractor-based neural network [9]. The following equations were adapted from Yildiz et al. (2013)[7]

$$\frac{dx^{(1)}}{dt}=\kappa_{1}[-Dx^{(1)}+W\tanh(x^{(1)})+I]+\varepsilon^{(1)}$$

$$I_{f}=\sum_{\gamma=1}^{8} \sum_{\omega=1}^{N_{syl}} \upsilon_{\gamma}^{(1)}\upsilon_{\omega}^{(1)}P_{f\gamma\omega}$$

Where

- x**^(1)^** is a vector for 6 frequency channels
- *D* is a diagonal self-connectivity matrix and *W* is an asymmetric synaptic connectivity matrix; designed to ensure that the Hopfield network has a global attractor whose location depends on vector *I* [7].
- ν_γ_^(1)^ and ν_ω_^(1)^ are the causal states for the gamma and syllable units, respectively
- P_fγω_ is the stored information about spectrotemporal patterns ST_fγω_ of syllables and is calculated with the following non-linear transformation.

$$P_{f\gamma\omega}=\sum_{i=1}^{6} D_{fi}ST_{i\gamma\omega}-\sum_{i=1}^{6} W_{fi}\tanh(ST_{i\gamma\omega})$$

The causal states of this level are predictions about spectrotemporal patterns of syllables and slow amplitude modulation.

$$\begin{matrix} \upsilon_{f}^{(0)}=x^{(1)}+\eta_{f}^{(1)} \\ \upsilon_{\text{A}}^{(0)}=\upsilon_{\text{A}}^{(1)}+\eta_{\text{A}}^{(1)} \end{matrix}$$

# Bibliography

1. Garofolo J, Lamel L, Fisher W, Fiscus J, Pallett D, Dahlgren N, et al. TIMIT Acoustic-Phonetic Continuous Speech Corpus LDC93S1. Web Download. Phila Linguist Data Consort. 1993; 1–94.

2. Chi T, Ru P, Shamma SA. Multiresolution spectrotemporal analysis of complex sounds. J Acoust Soc Am. 2005;118: 887–906. doi:10.1121/1.1945807

3. Hyafil A, Fontolan L, Kabdebon C, Gutkin B, Giraud AL. Speech encoding by coupled cortical theta and gamma oscillations. eLife. 2015;4: 1–45. doi:10.7554/eLife.06213

4. Hovsepyan S, Olasagasti I, Giraud A-L. Combining predictive coding and neural oscillations enables online syllable recognition in natural speech. Nat Commun. 2020;11: 3117. doi:10/gg2n6w

5. Ermentrout GB, Kopell N. Parabolic Bursting in an Excitable System Coupled with a Slow Oscillation. SIAM J Appl Math. 1986;46: 233–253.

6. Rabinovich MI, Varona P, Selverston AI, Abarbanel HDI. Dynamical principles in neuroscience. Rev Mod Phys. 2006;78: 1213–1265. doi:10.1103/RevModPhys.78.1213

7. Yildiz IB, von Kriegstein K, Kiebel SJ. From Birdsong to Human Speech Recognition: Bayesian Inference on a Hierarchy of Nonlinear Dynamical Systems. Jirsa VK, editor. PLoS Comput Biol. 2013;9: e1003219. doi:10.1371/journal.pcbi.1003219

8. Yildiz IB, Kiebel SJ. A hierarchical neuronal model for generation and online recognition of birdsongs. PLoS Comput Biol. 2011;7. doi:10.1371/journal.pcbi.1002303

9. Hopfield JJ. Neural Network and Physical Systems with Emergent Collective Computational Neural networks and physical systems with emergent collective computational abilities. 1982. doi:10.1073/pnas.79.8.2554
