# Supplementary figures and images for: Rhythmic modulation of prediction errors: A top-down gating role for the beta-range in speech processing
Source: PLoS Comput Biol. 2023 Nov 7;19(11):e1011595. doi: 10.1371/journal.pcbi.1011595 (PMC10655987; doi:10.1371/journal.pcbi.1011595)

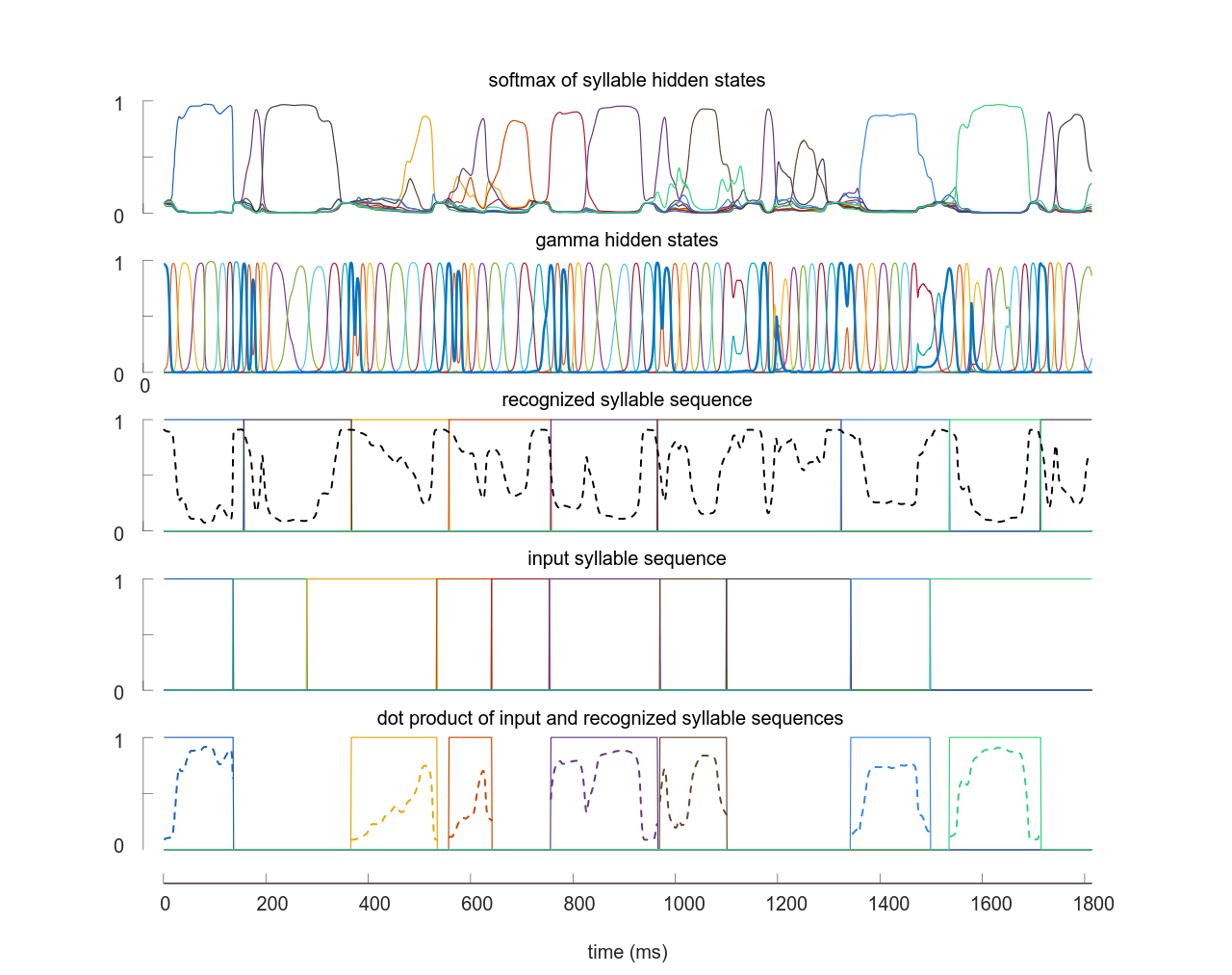

Supplement: S1 Fig — The top two panels represent the dynamics of the gamma and syllable hidden states during inference for an example sentence. For each subplot, colored lines were used to represent different gamma and syllable units. The gamma unit with a thick blue line corresponds to the first gamma unit, whose peak (amplitude more than 0.6) is used as a marker to indicate windows for identifying the “winner” syllable unit. For the latter, we look for the syllable unit with the highest average activation within a gamma window (time interval between two consecutive gamma 1 peaks). The sequence of the recognized syllables is shown in the 3rd panel (colored solid lines), whereas the dashed line indicates the entropy associated with the softmax of syllable hidden states (top panel). The sequence and duration of the syllables in the input are shown in the 4th subpanel. The model performance (the overlap metric) is evaluated with the sum of the dot-product (bottom subpanel) of recognized and input syllable sequences (subpanels 3 and 4) divided by the duration of the input sentence. The higher/closer to 1, the better the model is able to infer identity and duration of syllables in the input sentence. The overlap metric that also incorporates (Fig 4B) the entropy is calculated based on the sum of the dot product of recognized syllable sequence (solid lines on the 3rd panel), 1-entropy (the dashed line on the 3rd panel) and syllable sequence in the input (4th panel). (TIF) [file pcbi.1011595.s002.tif]

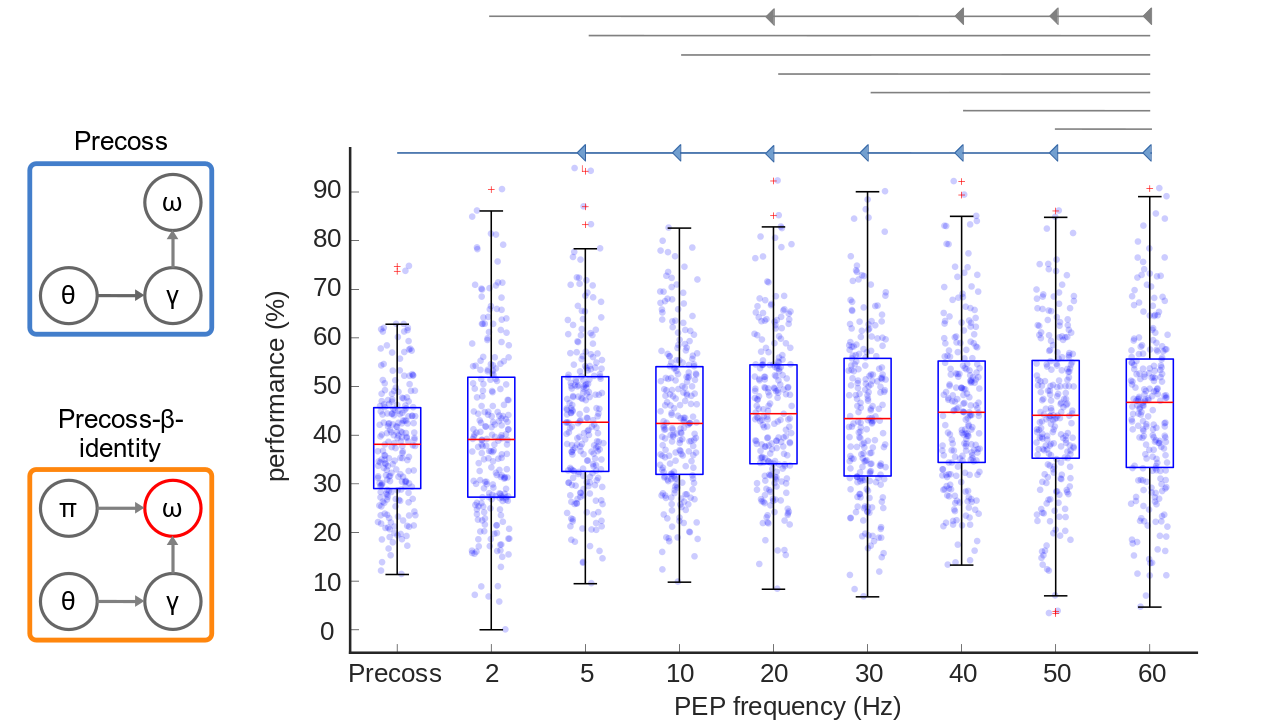

Supplement: S2 Fig — Simulation results on 220 sentences. Performance is evaluated based on the overlap between the recognized syllable sequence and the sequence of syllables in the input sentence (for details, see S1 Fig). We compare the performance of Precoss-β for different frequency values of PEP. For all frequencies, performance is better than that of Precoss with stationary precisions (S1 Table). Friedman test (χ2 = 24.77, p = 0.0008) indicated an effect of PEP frequency on model performance. Post-hoc pairwise comparisons (Bonferroni-corrected, S4 Table), indicated that performance of Precoss-β increased with frequency up to 5 Hz and reached a plateau (there is no statistically significant difference in the model’s performance for frequencies higher or equal to 5 Hz). Each point on the scatter plot represents the model performance in each sentence for the corresponding PEP frequency. The central-red mark of the box plots indicates the median, whereas bottom and top edges represent 25th and 75th percentiles. Red crosses indicate outliers, whereas whiskers extend to the highest and lowest overlap values that are not considered outliers. The blue line at the top represents comparisons of Precoss-β with Precoss, while triangular grey lines indicate comparisons within Precoss-β for different PEP frequencies. Arrows on these lines indicate significant differences, while the direction of the arrows indicates the sign of the effect. (TIF) [file pcbi.1011595.s003.tif]

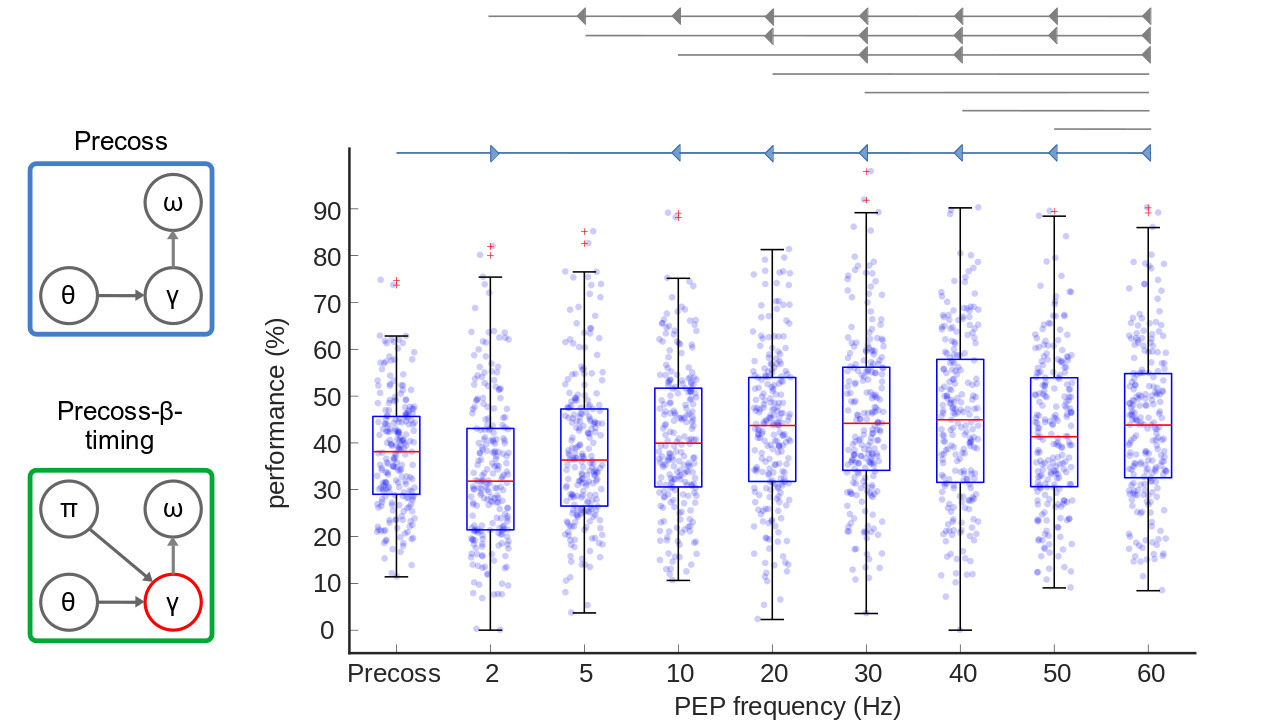

Supplement: S3 Fig — Simulation results on 220 sentences are presented in the figure. Performance is evaluated based on the overlap between the recognized and input syllable sequences (for details, see S1 Fig). Precoss-β outperforms Precoss for PEP frequencies higher or equal to 10 Hz, whereas for smaller frequencies the performance is worse (S2 Table). Friedman test (χ2 = 125.4, p = 5.727e-24) indicated an effect of PEP frequency on model performance. Post-hoc, multiple comparisons tests (corrected with Bonferroni procedure, S5 Table) indicated that Precoss-β performance increases with frequency and reaches a plateau at around 20 Hz. Each point on the scatter plot represents the value for each sentence for the corresponding PEP frequency. The central-red mark of the box plots corresponds to the median, whereas bottom and top edges represent 25th and 75th percentiles, respectively. Red crosses indicate outliers, whereas whiskers extend to the highest and lowest performance values that are not considered outliers. The blue line at the top represents comparisons of Precoss-β with Precoss, while triangular grey lines indicate comparisons between different PEP frequencies within Precoss-β. Arrows on these lines indicate significant differences, while the direction of the arrows indicates the sign of the effect. (TIF) [file pcbi.1011595.s004.tif]

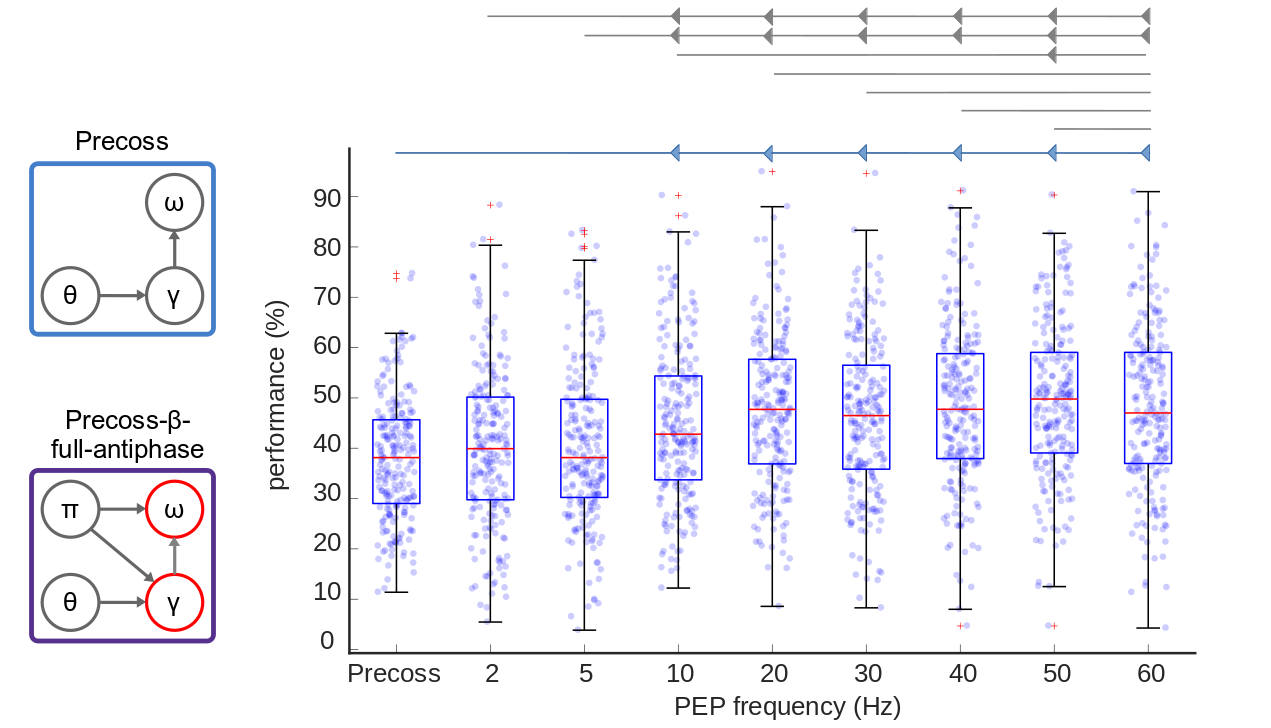

Supplement: S4 Fig — Simulation results on 220 sentences are presented in the figure. Performance is evaluated based on the overlap duration between the recognized syllable sequence and the sequence of syllables in the input sentence (for details, see S1 Fig). For this condition performance of Precoss-β is better than the performance of Precoss, with stationary precisions for all frequency values of the precision units (S3 Table). Friedman test (χ2 = 128.41.86, p = 1.351e-24) confirmed that the frequency of PEP affects model performance. Post-hoc, Bonferroni corrected pairwise comparisons indicated that the model performance increases with the frequency and reaches a plateau at 20 Hz (there are no statistically significant differences in performance for higher PEP frequencies, S6 Table). The central-red mark of the box plots corresponds to the median, whereas bottom and top edges represent 25th and 75th percentiles, respectively. Red crosses indicate outliers, whereas whiskers extend to the highest and lowest model performance values that are not considered outliers. The blue line at the top represents comparisons of Precoss-β with Precoss, while triangular grey lines indicate comparisons within Precoss-β for different PEP frequencies. Arrows on these lines indicate significant differences, while the direction of the arrows indicates the sign of the effect. (TIF) [file pcbi.1011595.s005.tif]

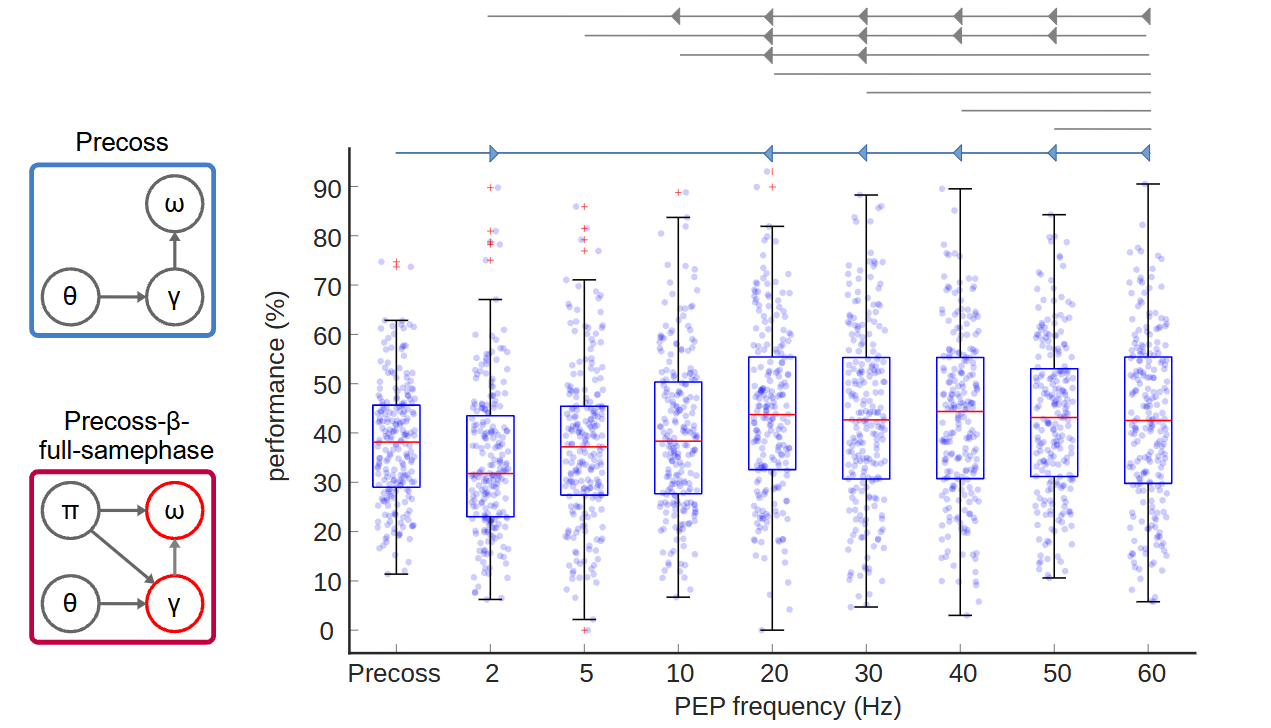

Supplement: S5 Fig — Simulation results on 220 sentences are presented in the figure. Performance is evaluated based on the overlap duration between the recognized syllable sequence and the sequence of syllables in the input sentence (for details, see S1 Fig). For this condition performance of Precoss-β is better than the performance of Precoss, with stationary precisions for all frequency values of the precision units (S3 Table). Friedman test (χ2 = 94.94.86, p = 1.192e-17) confirmed that the frequency of PEP affects model performance. Post-hoc, Bonferroni corrected pairwise comparisons indicated that the model performance increases with the frequency and reaches a plateau at 20 Hz (there are no statistically significant differences in performance for higher PEP frequencies, S6 Table). The central-red mark of the box plots corresponds to the median, whereas bottom and top edges represent 25th and 75th percentiles, respectively. Red crosses indicate outliers, whereas whiskers extend to the highest and lowest model performance values that are not considered outliers. The blue line at the top represents comparisons of Precoss-β with Precoss, while triangular grey lines indicate comparisons within Precoss-β for different PEP frequencies. Arrows on these lines indicate significant differences, while the direction of the arrows indicates the sign of the effect. (TIF) [file pcbi.1011595.s006.tif]

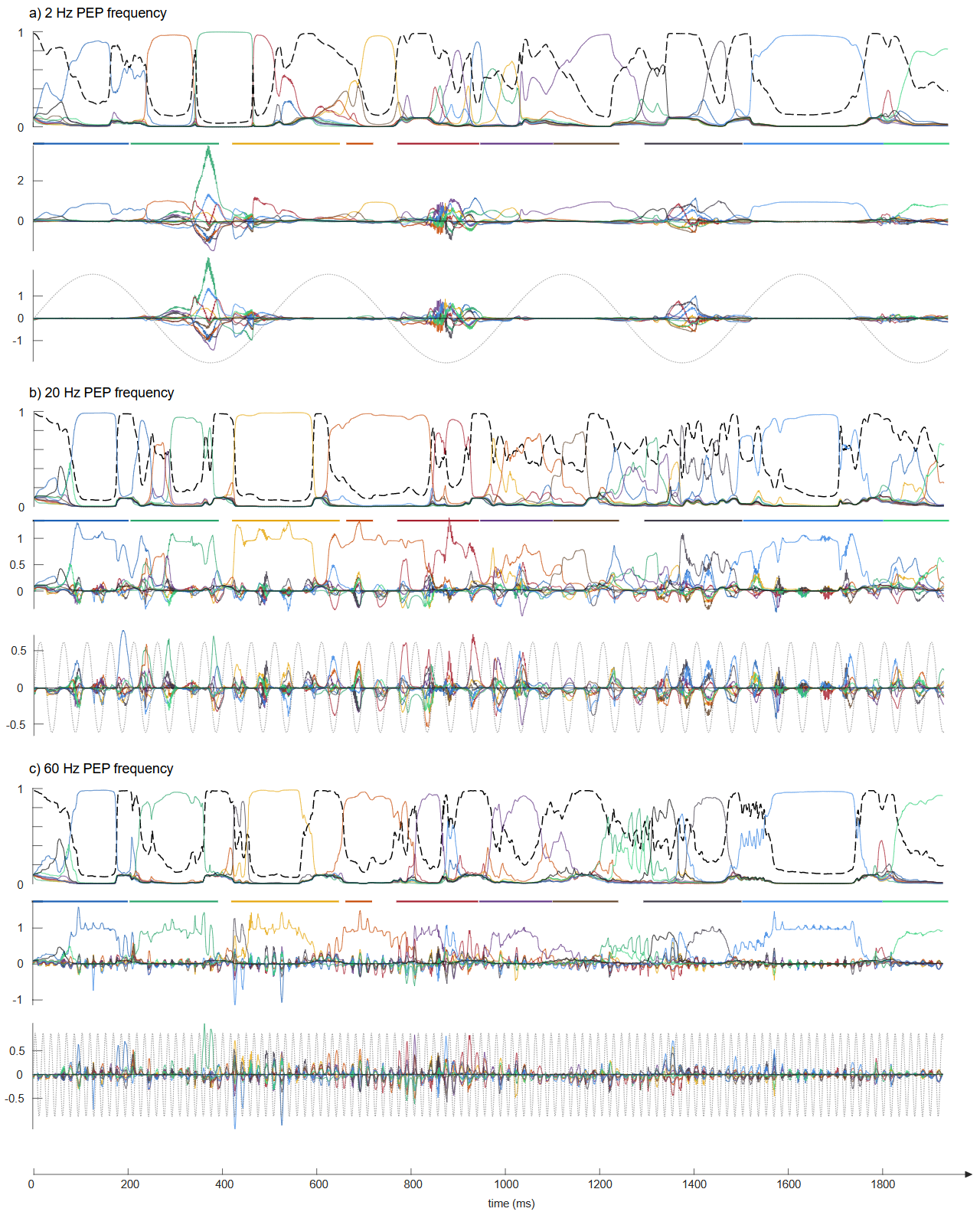

Supplement: S6 Fig — Each panel in the figure represents the effect of PEP on the dynamics of syllable recognition for different PEP frequencies. Each panel contains 3 plots. The top one represents the softmax of syllable hidden states (colour coded for different syllables in the input sentence), with the dashed line representing the entropy associated with the accumulated evidence. The middle plot shows the syllable hidden states, with the horizontal the bars representing the syllable sequence (identity and duration) in the input sentence. The bottom plot represents the bottom-up prediction errors for the syllable units, with the dashed line corresponding to the oscillation controlling the precision of the prediction errors. This comparison illustrates that during low PEP frequencies low/high precision phase spans often extend over several syllables in the input. This means that for many syllables the model is not able to integrate and accumulate sensory information. In case of higher PEP frequencies there is "always" unexplained prediction errors, that results in more noisy dynamics of syllable causal states. (TIF) [file pcbi.1011595.s007.tif]

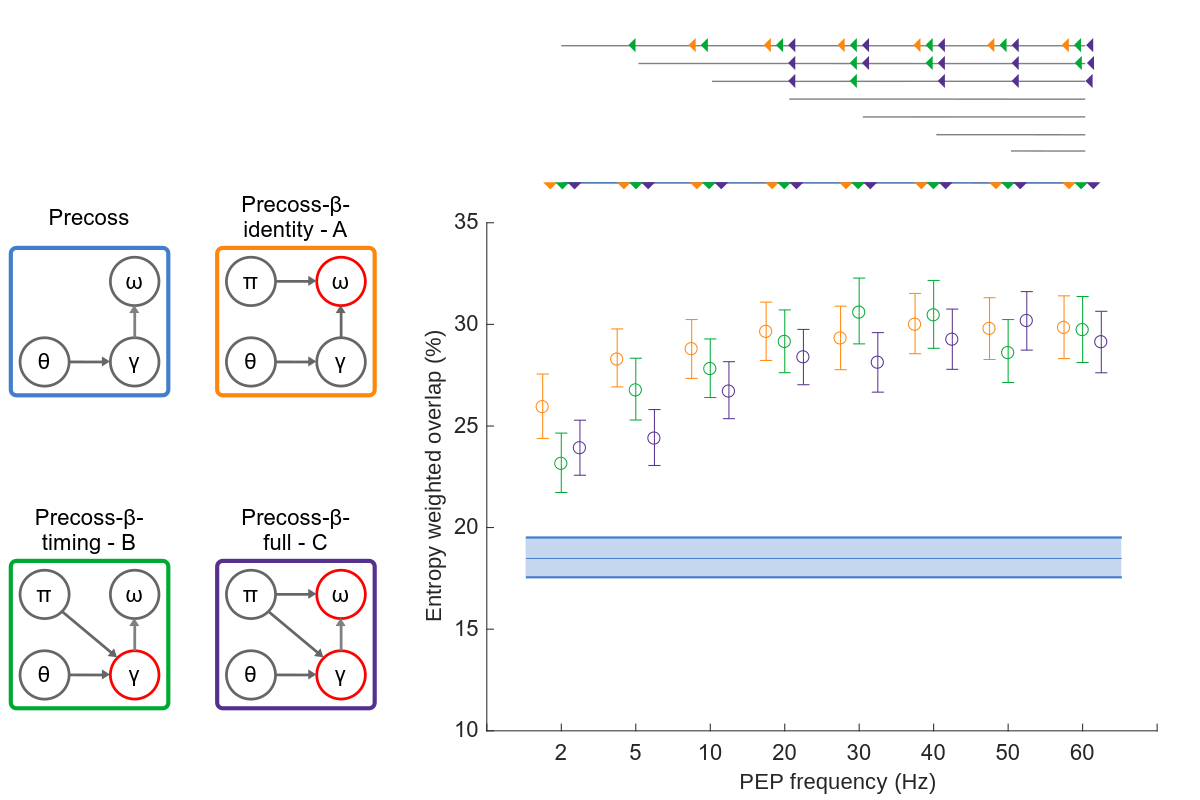

Supplement: S7 Fig — The figure illustrates the model performance based on the entropy weighted overlap metric (S1 Fig). The graph shows the mean performance and 95% confidence interval after bootstrapping for different Precoss-beta variants (color coded) and Precoss with fixed precision (the blue band). Arrows at the top indicate significant differences within model comparisons for different PEP frequencies. The direction of an arrow indicates the direction of the effect. Similarly, the blue line and the arrows on it show the comparisons for each Precoss-beta variant versus Precoss, with the direction of the arrows indicating that Precoss-beta with oscillating precisions outperforms Precoss for all variants and PEP frequencies. (TIF) [file pcbi.1011595.s008.tif]

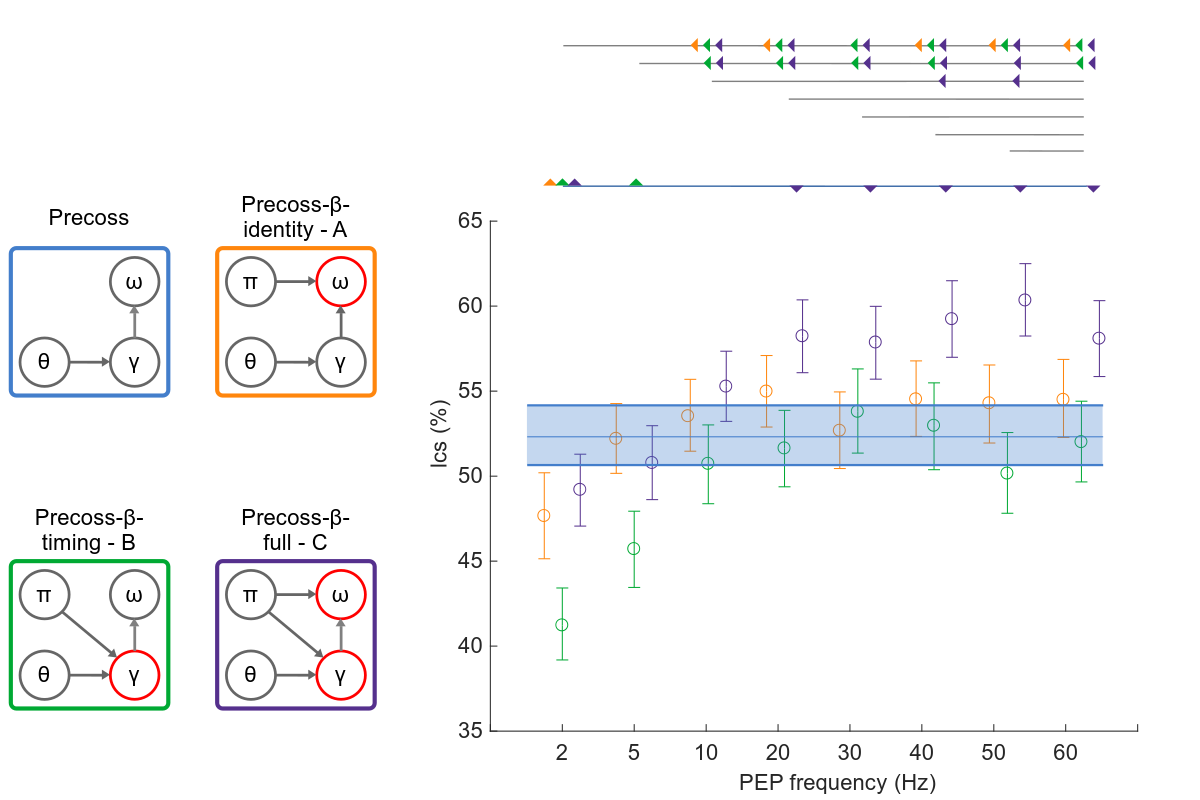

Supplement: S8 Fig — The figure illustrates the evaluation of the model variants based on the longest-common-sub-sequence (lcs). For each sentence, the lcs between the recognized syllable sequence and the syllable sequence in the input sentence is retrieved. The length of the lcs is divided by the number of syllables in the input sentence, giving the percentage on which this figure is based. Thus, for each model variant (color coded) and for each PEP frequency, we show the mean lcs (in %) and the 95% confidence interval. The arrows (color coded) represent the statistically significant differences for within-model comparisons for different PEP frequency values. The direction of the arrows represents the direction of the effect (pointing to the left would mean that the frequency on the left has a statistically lower lcs value than the frequency on the right). Similarly, the blue line and the arrows on it represent the comparison between Precoss and Precoss-beta variants, where the arrows pointing downwards indicate that the corresponding Precoss-beta variant (color coded) and frequency has significantly better performance. (TIF) [file pcbi.1011595.s009.tif]
